# Alternative exon usage in *TRIM21* determines the antigenicity of Ro52/TRIM21 in systemic lupus erythematosus

**DOI:** 10.1172/jci.insight.163795

**Published:** 2022-10-10

**Authors:** Eduardo Gomez-Bañuelos, M. Javad Wahadat, Jessica Li, Merlin Paz, Brendan Antiochos, Alessandra Ida Celia, Victoria Andrade, Dylan P. Ferris, Daniel W. Goldman, Erika Darrah, Michelle Petri, Felipe Andrade

**Affiliations:** 1 Division of Rheumatology, The Johns Hopkins University School of Medicine, Baltimore, Maryland, USA.; 2 Department of Immunology and; 3 Department of Paediatric Rheumatology, Sophia Children’s Hospital, Erasmus University Medical Center, Rotterdam, Netherlands.; 4 Rheumatology Unit, Department of Clinical Internal, Anaesthesiolagical and Cardiovascular Sciences, Sapienza University of Rome, Rome, Italy.

**Keywords:** Autoimmunity, Autoimmune diseases, Lupus, Neutrophils

## Abstract

The origin and mechanisms of autoantigen generation in systemic lupus erythematosus (SLE) are poorly understood. Here, we identified SLE neutrophils activated in vivo by IFN as a prominent source of Ro52, also known as tripartite motif–containing protein 21 (TRIM21), a critical autoantigen historically thought to be primarily generated by keratinocytes in SLE. Different from mononuclear cells and keratinocytes, SLE neutrophils are enriched in several unique Ro52 species containing a core sequence encoded by exon 4 (Ro52Ex4) in *TRIM21*. Ro52Ex4 is the main target of anti-Ro52 antibodies and is found in 2 Ro52 variants (Ro52α and an isoform termed Ro52γ) upregulated in SLE neutrophils. Further analysis of Ro52γ revealed a subset of autoantibodies against a unique C-terminal domain (Ro52γCT) generated from a frameshift due to the lack of exon 6 in Ro52γ. Antibodies to Ro52Ex4 and Ro52γCT distinguish SLE patient subsets characterized by distinct clinical, laboratory, treatment, and transcriptional profiles that are not discerned by the “classical” anti-Ro52 antibodies. These studies uncover IFN-activated neutrophils as a key source of unique immunogenic forms of Ro52 in SLE. Moreover, the finding of Ro52Ex4 and Ro52γCT as core targets of anti-Ro52 antibodies focus interest on Ro52γ as the potential isoform toward which immunological tolerance is initially lost in SLE.

## Graphical abstract

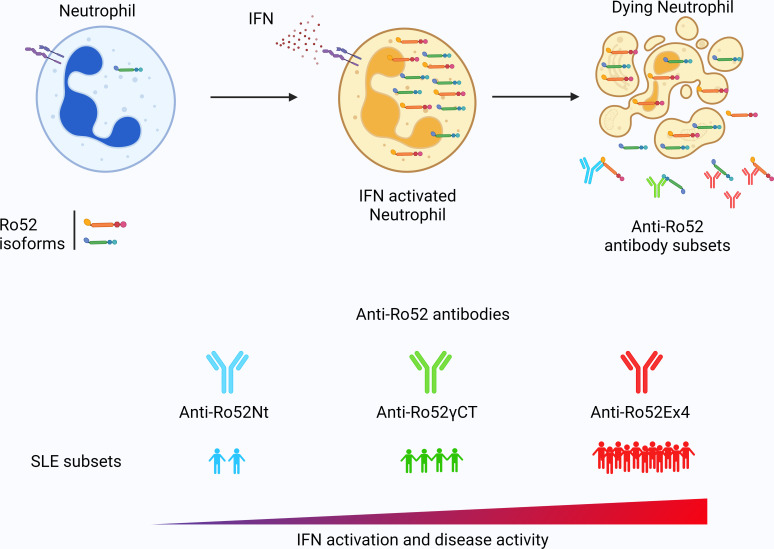

## Introduction

Ro52, also known as tripartite motif–containing protein 21 (TRIM21), is an IFN-induced E3 ubiquitin ligase that drives negative-feedback regulation during inflammation (1, 2) and has also been identified as a cytosolic antibody receptor involved in the intracellular clearance of antibody-coated viruses, such as adenovirus (3). Initially described as part of the Ro antigenic particle, Ro52 is among the first autoantigens discovered in systemic lupus erythematosus (SLE) (4, 5), a multisystemic autoimmune disease characterized by sustained IFN signaling and high-titer autoantibodies, leading to immune-mediated tissue damage (6, 7). Antibodies to Ro52 are frequently detected before clinical onset in SLE and are found in up to 40% of patients with established disease (1, 8).

Although Ro52 is largely expressed by immune cells under steady-state conditions (2, 9), its pathogenic relevance in SLE has been centered on keratinocytes, mainly because of the initial association between antibodies against the Ro particle with photosensitivity and cutaneous lupus (10–13). In particular, the redistribution of Ro52 on the cell surface and apoptotic blebs of keratinocytes in response to ultraviolet radiation is considered the main mechanism related to the immunogenic source of Ro52 in SLE (14, 15). Unexpectedly, during the search for in vivo IFN-induced autoantigens expressed by neutrophils in patients with SLE, we discovered Ro52 as a prominent neutrophil autoantigen with multiple structural forms, of which expression is related to in vivo IFN-induced activation in SLE neutrophils. Understanding the pathologic significance of Ro52 expression in SLE neutrophils is the focus of research addressed in the present study.

## Results

### Neutrophils express distinct forms of Ro52 linked to IFN activation in SLE.

**Figure 1:**
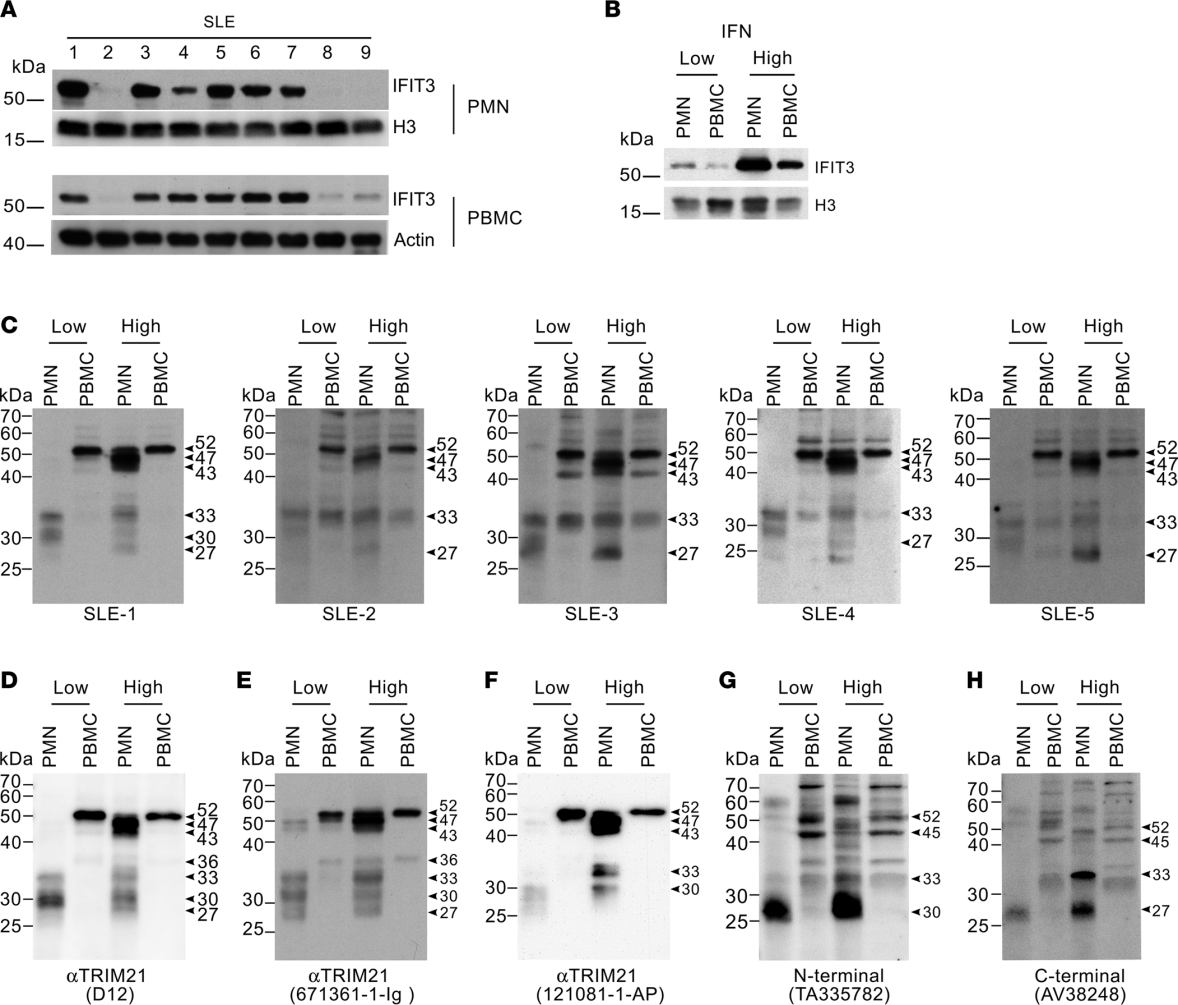
SLE neutrophils overexpress distinct forms of Ro52 in relation to IFN pathway activation. (**A**) Neutrophils (PMN) and PBMCs from 19 consecutive patients with SLE were analyzed by immunoblotting using antibodies to IFIT3, histone H3 (H3), and β-actin (loading controls). Representative samples from 9 patients are shown. (**B**–**H**) Lysates from SLE neutrophils and PBMCs with low and high activation by IFN based on IFIT3 expression (**B**) were used to screen 20 SLE sera (**C**) and 5 commercial antibodies to Ro52 (**D**–**H**). H3 is shown as loading control in **B**. In **C**, data from 5 SLE sera recognizing a set of autoantigens overexpressed in SLE neutrophils with high IFIT3 expression are shown.

While neutrophils have gained major interest as a source of interferogenic signals in SLE, including the release of genomic and oxidized mitochondrial DNA that activate plasmacytoid DCs (16–20), it is noteworthy that neutrophils are also important targets of IFN-I in this disease (16), suggesting the possibility that these cells may be an important source of autoantigens induced by IFN in SLE. To search for neutrophil autoantigens linked to IFN activation in SLE, we initially identified in vivo–activated cells by IFN through the study of protein expression of the IFN-induced protein with tetratricopeptide repeats 3 (IFIT3) — as marker of IFN-induced activation (21) — in freshly isolated peripheral blood neutrophils and peripheral blood mononuclear cells (PBMCs) from consecutive patients with SLE (Figure 1A).

SLE neutrophils and PBMCs with the lowest and highest IFIT3 expression (hereafter IFN-low and IFN-high, respectively) were then pooled according to cell type and IFIT3 expression (Figure 1B) and were used to screen SLE sera (*n =* 20) by immunoblotting to identify neutrophil autoantigens whose expression parallel that of IFIT3. From these studies, we initially focused our interest on 5 SLE sera detecting a common pattern of 3 bands of approximately 43, 47, and 52 kDa, which were highly expressed by IFN-high SLE neutrophils but absent in IFN-low SLE neutrophils (Figure 1C). Among these bands, the 52 kDa species was also expressed in PBMCs, regardless of IFN status (Figure 1C). Using 2-dimensional electrophoresis and mass spectrometry (MS), this set of bands was identified as Ro52 (Supplemental Figure 1; supplemental material available online with this article; https://doi.org/10.1172/jci.insight.163795DS1).

Using commercial antibodies, we further addressed whether Ro52 has unique patterns of expression in SLE neutrophils and PBMCs according to IFIT3 levels (Figure 1, D–H). Intriguingly, different patterns of Ro52 were detected depending on the commercial antibody utilized. Two mouse monoclonals and 1 rabbit polyclonal antibody to Ro52, in which the target epitopes are not disclosed (Figure 1, D–F), reproduced identical patterns of Ro52 detected in neutrophils and PBMCs by SLE serum (Figure 1C), including some additional bands of approximately 27–33 kDa found in neutrophils. In contrast, 2 commercial antibodies against the N-terminal and C-terminal regions (amino acid residues 51–100 and 421–470, respectively) showed detection of Ro52 — albeit modest — in PBMCs and poor detection of the set of Ro52 bands enriched in IFN-high neutrophils (Figure 1, G and H). Considering that the antibodies are directed against different regions in Ro52, we hypothesized that these discrepancies may be explained by the presence of distinct structural forms of Ro52 — either transcriptional variants, degradation products, or both.

### SLE neutrophils express splicing variants of Ro52.

**Figure 2:**
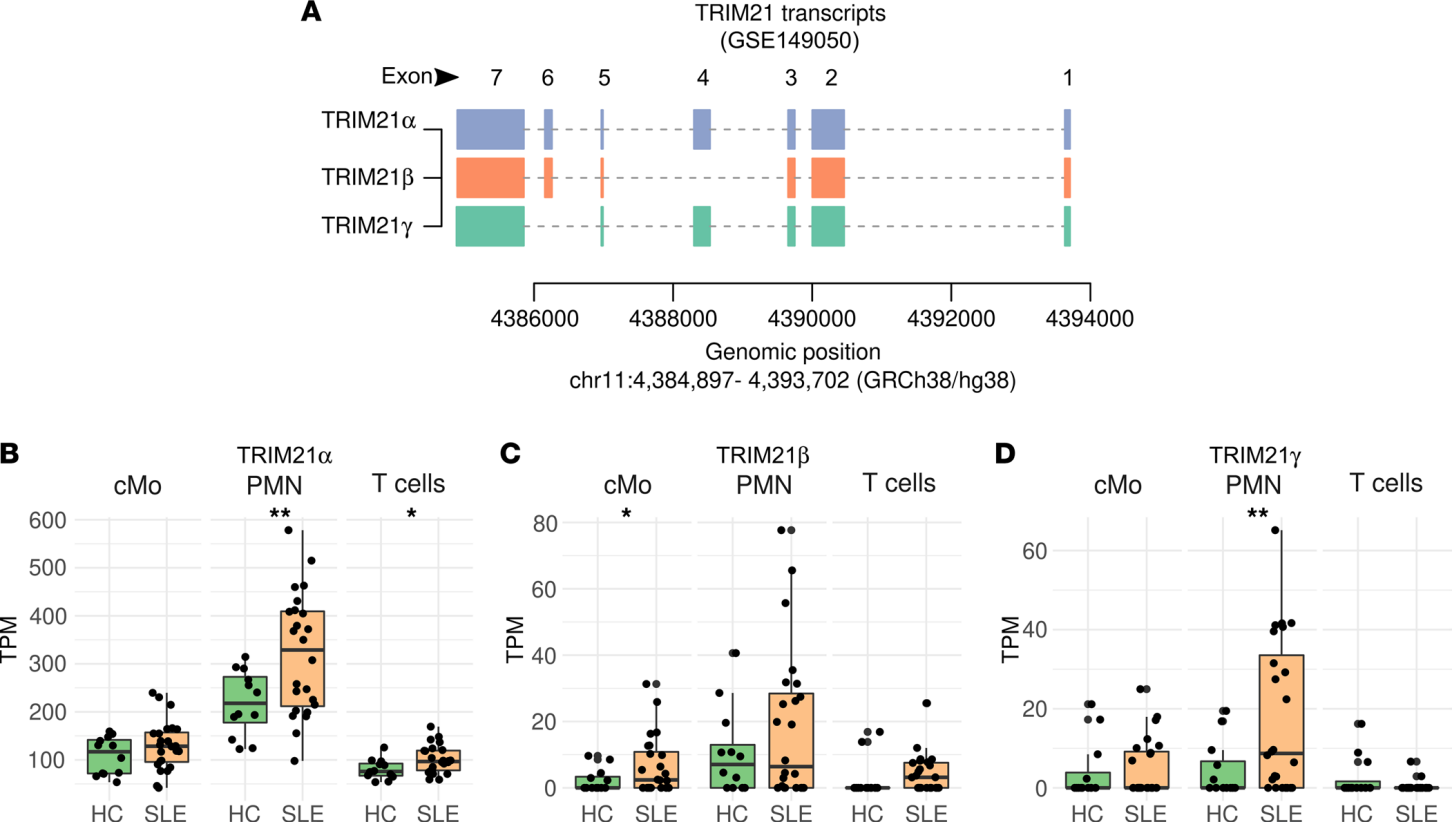
Neutrophils express splicing variants of TRIM21/Ro52. (**A**) Schematic representation of the transcripts corresponding to TRIM21 isoforms found in a publicly available RNA-Seq data set (GSE149050) from classical monocytes (cMo), neutrophils (PMN), and T cells from patients with SLE (*n =* 24) and healthy controls (HC, *n =* 12) using the ‘new tuxedo’ pipeline. Each solid block represents an exon. (**B**–**D**) Differential expression analyses of TRIM21α (**B**), TRIM21β (**C**), and TRIM21γ (**D**) between HC and SLE according to cell type. Pairwise comparisons between HC and SLE were done using Wilcoxon’s test. *P < 0.05, **P < 0.01.

To gain insight into the source of the different Ro52 species found in IFN-high SLE neutrophils, we focused on the search for Ro52 splicing variants. Ro52 is encoded by the *TRIM21* gene, which is split into 7 exons (Figure 2A). The 5′ untranslated sequence is divided between exons 1 and 2, and the initiation codon is located in exon 2 (22). In order to identify Ro52 isoforms, we analyzed publicly available RNA-Seq data sets (GSE149050 and GSE124939) of 3 circulating immune cell types (including neutrophils, classical monocytes [cMo], and T cells) (23) and keratinocytes from patients with SLE (24). Using the ‘new Tuxedo’ pipeline (25), we identified 3 TRIM21 transcripts in circulating cells: 1 corresponding to the sequence encoding the full-length protein (termed TRIM21α/Ro52α), a second transcript corresponding to TRIM21β/Ro52β (resulting from the splicing of exon 3 to exon 5, skipping exon 4) (22), and a variant termed TRIM21γ/Ro52γ that results from the alternative splicing of exon 5 to exon 7, skipping exon 6 (Figure 2A).

This analysis also demonstrated that the expression of Ro52 variants varies depending on the cell type. Thus, compared with controls, SLE T cells and cMo showed a significant upregulation of the Ro52α and Ro52β transcripts, respectively (Figure 2, B and C), while SLE neutrophils were distinguished by a significant upregulation of both Ro52α and Ro52γ transcripts (Figure 2, B and D, respectively). In contrast to circulating cells in SLE, keratinocytes only expressed TRIM21α/Ro52α and a variant termed TRIM21δ/Ro52δ that results from the alternative splicing of exon 6 to an internal splicing site in exon 7 (Supplemental Figure 2). Interestingly, even though SLE keratinocytes are hyperresponsive to IFNa2 (24), the expression of TRIM21α/Ro52α and TRIM21δ/Ro52δ was not different in SLE compared with healthy control keratinocytes either at baseline or after IFN stimulation (Supplemental Figure 2).

### Autoantibodies against Ro52 target a sequence encoded by exon 4 in TRIM21.

**Figure 3:**
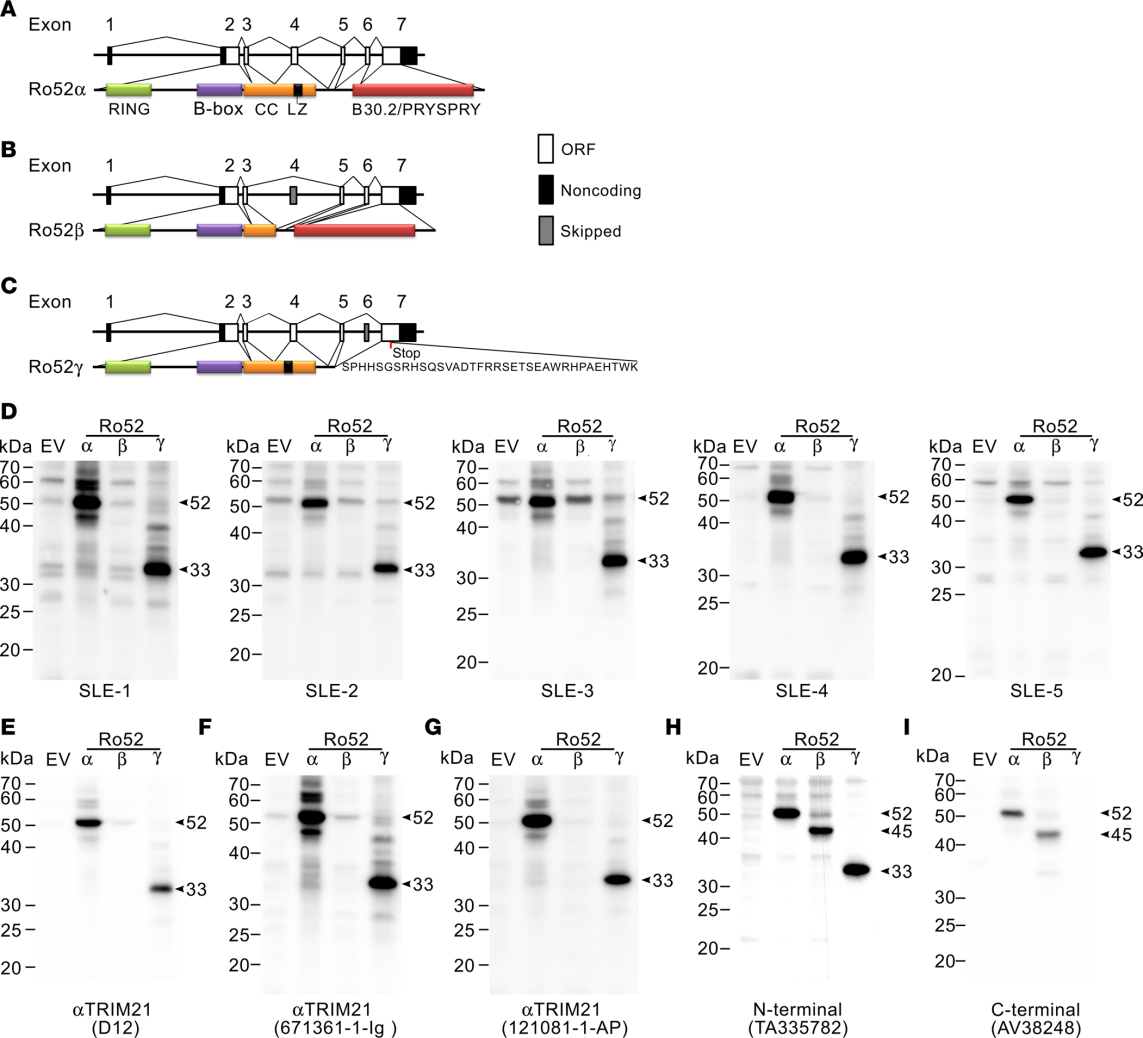
Autoantibodies against Ro52 target a sequence encoded by exon 4 in *TRIM21*. (**A**–**C**) Schematic representation of exon usage and structural domains in Ro52 isoforms. (**D**–**I**) SLE sera (**D**) and commercial anti-Ro52 antibodies (**E**–**I**) from Figure 1, C–H, were used to immunoblot cell lysates from HEK293 cells transfected with mock (empty vector [EV]) or plasmids expressing Ro52α, Ro52β, or Ro52γ. The arrows denote the Ro52 isoforms. Detection of Ro52α, Ro52β, and Ro52γ by the N-terminal anti-Ro52 antibody in **H** is also shown as a loading control. RING, really interesting new gene; BB, B-box domain; CC, coiled-coil; LZ, leucine zipper.

To investigate whether the distinct patterns of Ro52 detection by SLE sera and commercial antibodies in SLE leukocytes is explained by targeting unique Ro52 isoforms, we focused on splicing variants upregulated in circulating SLE cells. Ro52α, which corresponds to full-length Ro52, is a molecule of 52 kDa consisting of 475 amino acid residues (22). The deduced structure contains 3 distinct domains. The amino-terminal region includes 2 zinc-finger motifs (a really interesting new gene–finger [RING-finger] and a B-box), followed by a coiled-coil stretch including a putative leucine zipper and a B30.2/PRYSPRY domain in the C-terminal end (Figure 3A) (1). Ro52β is an approximately 45 kDa protein that lacks the sequence encoded by exon 4 (amino acid residues 167–245), which includes the leucine zipper and part of the coiled-coil domain (Figure 3B) (22). Ro52γ is a variant consisting of a C-terminally truncated protein lacking the B30.2/PRYSPRY domain. The predicted product is a protein of 287 amino acids (~33 kDa) containing the RING, B-box, and coiled-coil domains, followed by a C-terminal sequence of 35 amino acid residues generated from a frameshift at the junction of exons 5 and 7 (Figure 3C).

To address the detection of Ro52 isoforms by SLE sera and commercial anti-Ro52 antibodies, cell lysates from HEK293 cells transfected to express the different Ro52 variants were analyzed by immunoblotting. Strikingly, all SLE sera and commercial antibodies recognizing the set of Ro52 bands in IFN-high SLE neutrophils (Figure 1, C–F) were specific for Ro52α and Ro52γ (Figure 3, D–I), demonstrating that the main epitope targeted by antibodies against the Ro52 neutrophil variants is located within a sequence encoded by exon 4 (hereafter Ro52Ex4), which is missing in Ro52β. Since Ro52β contains both the N-terminal and C-terminal domains (Figure 3B), it is likely that the ~45 kDa band detected in PBMCs by antibodies to the N-terminal and C-terminal regions of Ro52 (Figure 1, G and H, respectively) corresponds to Ro52β, which is consistent with the upregulation of its transcript in SLE monocytes (Figure 2C). These data also suggest that Ro52β protein is not detected in neutrophils.

**Figure 4:**
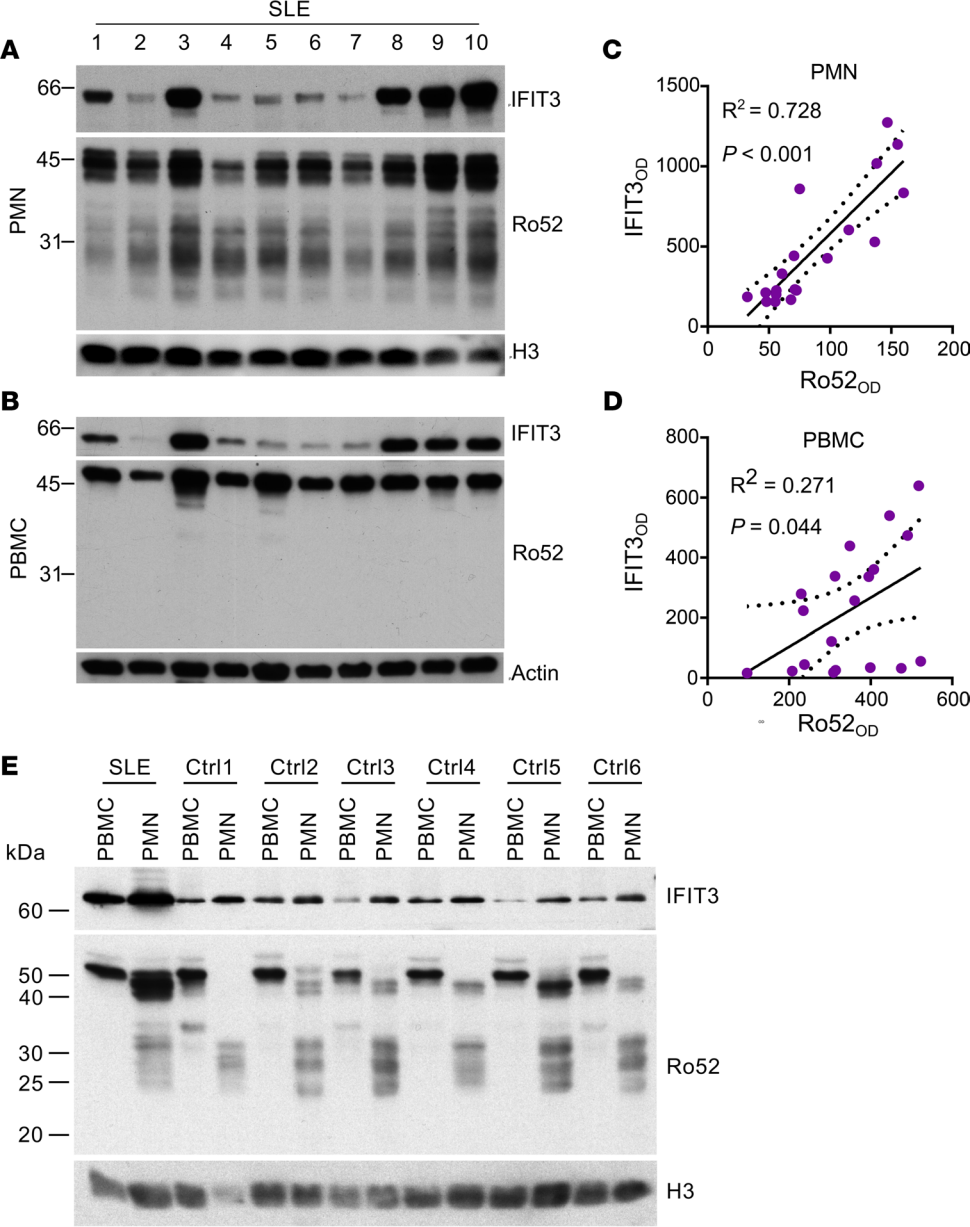
Relationship between IFIT3 and Ro52 expression in neutrophils and PBMCs from patients with SLE and healthy controls. (**A** and **B**) Neutrophils (PMN) and PBMCs from 19 consecutive patients with SLE were analyzed by immunoblotting using antibodies to IFIT3, Ro52 (D-12 mouse monoclonal antibody), histone H3 (H3), and β-actin (loading controls). Representative data from 10 patients are shown. (**C** and **D**) Correlation between the expression of IFIT3 and Ro52 in PMN and PBMCs. The expression of Ro52 and IFIT3 was quantified by densitometry from the corresponding bands in **A** and **B**, and the values were fitted to a linear regression model. (**E**) PBMCs and PMN from 12 healthy controls (Ctrl) and 1 patient with SLE were analyzed by immunoblotting using antibodies to IFIT3, Ro52 (D-12 mouse monoclonal antibody), and H3 (loading control). Representative data from 6 healthy controls are shown. The SLE samples were included for comparison.

Using the monoclonal antibody D-12 (Figure 1D), we further demonstrated that neutrophils from consecutive patients with SLE are highly enriched with Ro52 species containing the Ro52Ex4 epitope. Furthermore, these Ro52 species exhibited a strong correlation with IFIT3 protein expression (*R*^2^ = 0.728, *P <* 0.001) (Figure 4, A and C). In contrast, a single band of Ro52 kDa was detected in SLE PBMCs, which also correlated — albeit less strongly— with IFIT3 levels (*R*^2^ = 0.271, *P* = 0.044) (Figure 4, B and D). In cells from healthy donors, however, although the Ro52 kDa species of Ro52 was prominent in PBMCs, it was absent in neutrophils (Figure 4E). Moreover, the 43 kDa and 47 kDa species showed variable expression in control neutrophils, and this expression was minimal compared with SLE neutrophils (Figure 4E). The set of approximately 27–33 kDa bands were present both in healthy and SLE neutrophils (Figure 4E). Together, these data demonstrate that, compared with PBMCs and healthy control neutrophils, SLE neutrophils are highly enriched with Ro52 species containing the Ro52Ex4 sequence, whose expression correlates with the IFN-induced marker IFIT3.

### Splicing variation of exons 4 and 6 in TRIM21 determines the antigenic targets of autoantibodies to Ro52 in SLE.

**Figure 5:**
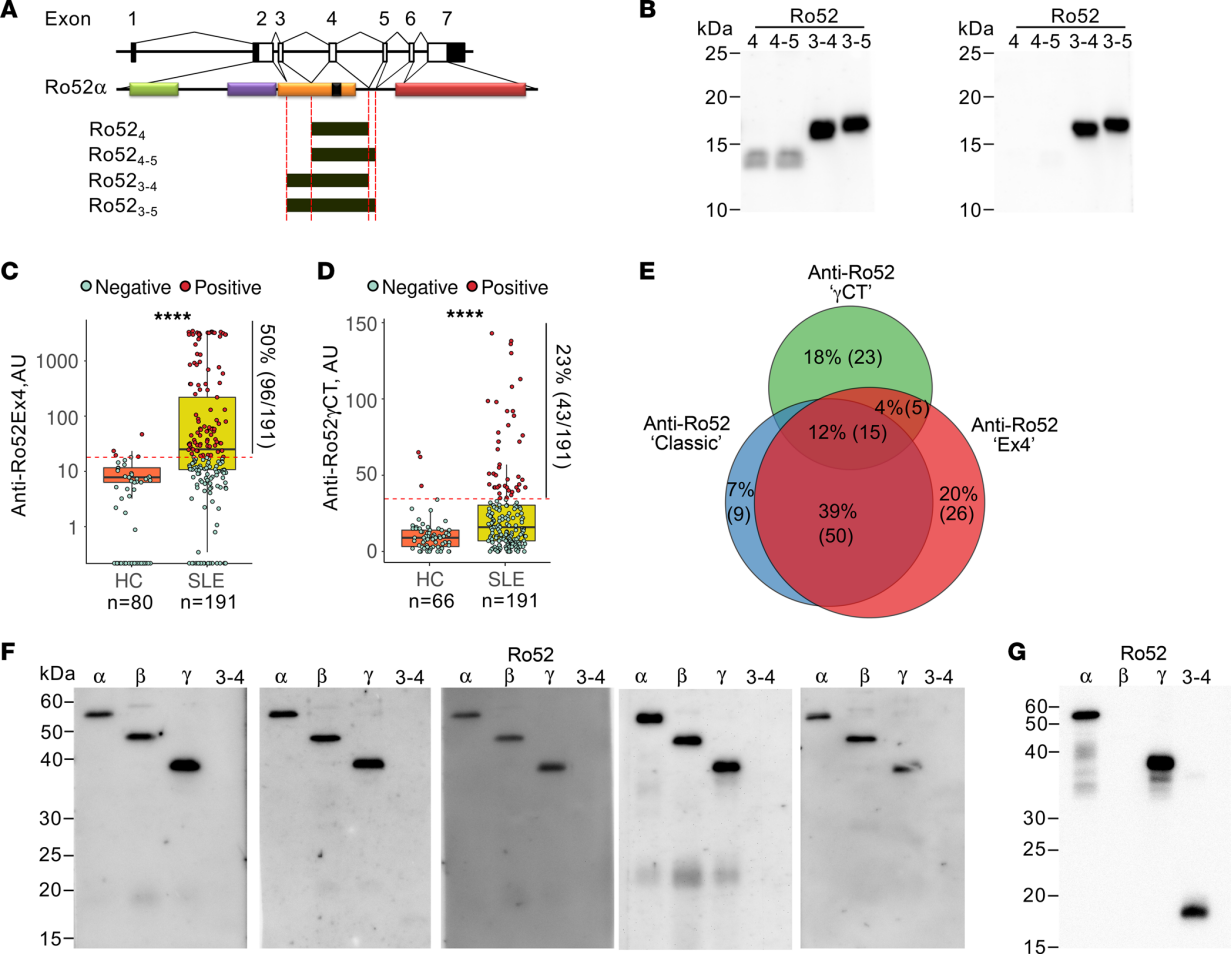
Patients with SLE have autoantibodies targeting distinct Ro52 isoforms. (**A**) Schematic representation showing the regions encoded by *TRIM21* exon 4, exons 4 and 5, exons 3 and 4, and exons 3–5 in Ro52. (**B**) Recombinant proteins containing the sequence encoded by *TRIM21* exon 4, exons 4 and 5, exons 3 and 4, and exons 3–5 were detected by immunoblotting using SLE sera positive for anti-Ro52Ex4 antibodies. Representative data from 6 sera are shown. (**C** and **D**) Levels of antibodies to Ro52Ex4 (**C**) and Ro52γCT (**D**) in sera from the SPARE cohort (SLE) and healthy controls (HC). Comparisons were done using Student’s *t* test. (**E**) Venn diagram depicting the anti-Ro52 antibody intersections (overlap) in 128 of 191 patients with SLE positive for anti-Ro52Ex4, anti-Ro52γCT, and/or anti-Ro52‘classic’ antibodies. (**F** and **G**) Recombinant Ro52α, Ro52β, and Ro52γ and the sequence encoded by Ro52 exons 3 and 4 were used to analyze by immunoblotting SLE sera from the none overlapping anti-Ro52‘classic’ antibodies (*n =* 9) (representative data from 5 sera are shown) (**F**) and anti-Ro52Ex4 serum (**G**). ****P < 0.0001.

Although Ro52Ex4 is well detected by SLE sera when found in the context of Ro52α and Ro52γ (Figure 3D), we further addressed whether the isolated Ro52Ex4 sequence is sufficient for efficient antibody recognition. Thus, we generated recombinant proteins containing the Ro52Ex4 amino acid sequence either alone or in combination with the sequences encoded by flanking exons 3 and/or 5 — i.e., exon 4, exons 4 and 5, exons 3 and 4, and exons 3–5 (amino acid residues 167–245, 167–253, 137–245, and 137–253, respectively) (Figure 5A) — and tested their recognition by SLE sera using immunoblotting (Figure 5B). Although Ro52Ex4 is the target of SLE sera in Ro52α and Ro52γ (Figure 3D), the presence of exon 3 importantly enhanced or was necessary for the efficient detection of the isolated Ro52Ex4 protein sequence (Figure 5B). Since the lack of recognition of Ro52β by SLE sera excludes the possibility that the region encoded by exon 3 is independently immunogenic in SLE (Figure 3D), it is likely that the exon 3–encoded sequence facilitates antibody binding by stabilizing the epitope encoded by exon 4. Alternatively, it is possible that the sequence encoded at the junction between exons 3 and 4 may play some role in autoantibody recognition.

Since the region encoded by exon 3 enables efficient detection of antibodies to Ro52Ex4 (i.e., anti-Ro52Ex4 antibodies), we used the recombinant protein containing amino acid residues 137–245 to screen for antibodies in SLE sera from the “Study of biological Pathways, Disease Activity and Response markers in patients with Systemic Lupus Erythematosus” (SPARE) lupus cohort — for which extensive clinical and serologic variables are available (26, 27) — and 80 healthy controls. Demographic, clinical, and laboratory features of the SLE cohort are summarized in Supplemental Table 1. Patients with SLE had significantly elevated serum levels of anti-Ro52Ex4 antibodies compared with healthy controls (*P* < 0.0001). Using a cut-off value determined by ROC curve analysis, 50% of patients with SLE (96 of 191) and 5% of controls (4 of 80) were positive for anti-Ro52Ex4 antibodies (Figure 5C).

Since Ro52Ex4 is found in variants Ro52α and Ro52γ, which are both transcriptionally upregulated in SLE neutrophils (Figure 2, B and D), either isoform could serve as the antigen driving the production of anti-Ro52Ex4 antibodies in SLE. Nevertheless, it is intriguing that autoantibodies against the C-terminal half of Ro52α (amino acid residues 268–475, also shared by Ro52β) are rare (i.e., 0%) among anti-Ro52 antibodies in SLE (28). While it is possible that the canonical C-terminal region of Ro52α is not a self-immunogen in SLE, it is also possible that the Ro52 variant responsible for the production of antibodies against Ro52Ex4 does not contain the classic C-terminal sequence. This focused our attention on Ro52γ, which differs from Ro52α in that it possesses a unique C-terminal sequence caused by a frameshift at the junction of exons 5 and 7. Following the same principle that other regions in self-immunogenic Ro52 variants should be targeted by autoantibodies in SLE, we looked for antibodies against the unique C-terminal sequence found in Ro52γ (hereafter Ro52γCT). Importantly, a Basic Local Alignment Search Tool (BLAST) search for this sequence against the catalog of annotated eukaryotic and prokaryotic proteins showed no similarity to any other known protein. Antibodies to Ro52γCT were significantly increased in SLE compared with healthy controls (*P <* 0.001) and found in 22.5% (43 of 191) of patients with SLE and 3.8% (3 of 80) healthy controls (Figure 5D).

In the SPARE cohort, 39% (74 of 191) of the patients have antibodies to Ro52 (hereafter anti-Ro52‘classic’ antibodies) as detected by the clinically available assay (Supplemental Table 1). Analysis of the intersection of the different anti-Ro52 antibodies revealed that 88% (65 of 74) of patients with SLE positive for anti-Ro52‘classic’ antibodies are also positive for anti-Ro52Ex4 antibodies (Figure 5E), demonstrating that positivity to anti-Ro52Ex4 antibodies identifies the majority of anti-Ro52‘classic’ antibodies in SLE. Further analysis of the small subset of SLE sera positive for anti-Ro52‘classic’ antibodies, but negative for antibodies to Ro52Ex4 (*n =* 9), revealed that these sera target the N-terminal sequence (i.e., anti-Ro52Nt) shared by Ro52α, -β, and -γ (Figure 5F). This pattern of detection importantly contrasts with anti-Ro52Ex4 serum, which only recognizes Ro52α and Ro52γ and the RoEx3-4 protein sequence (Figure 5G). Thus, antibodies to Ro52Nt represent 4.7% (9 of 191) of anti-Ro52 antibodies in the SLE cohort, and this is consistent with a previous study showing that 4% of anti-Ro52–positive SLE sera recognize the N-terminal sequence in Ro52 (amino acid residues 1–127) (28).

### Antibodies targeting unique regions in Ro52 distinguish distinct clinical subsets and disease activity in SLE.

**Figure 6:**
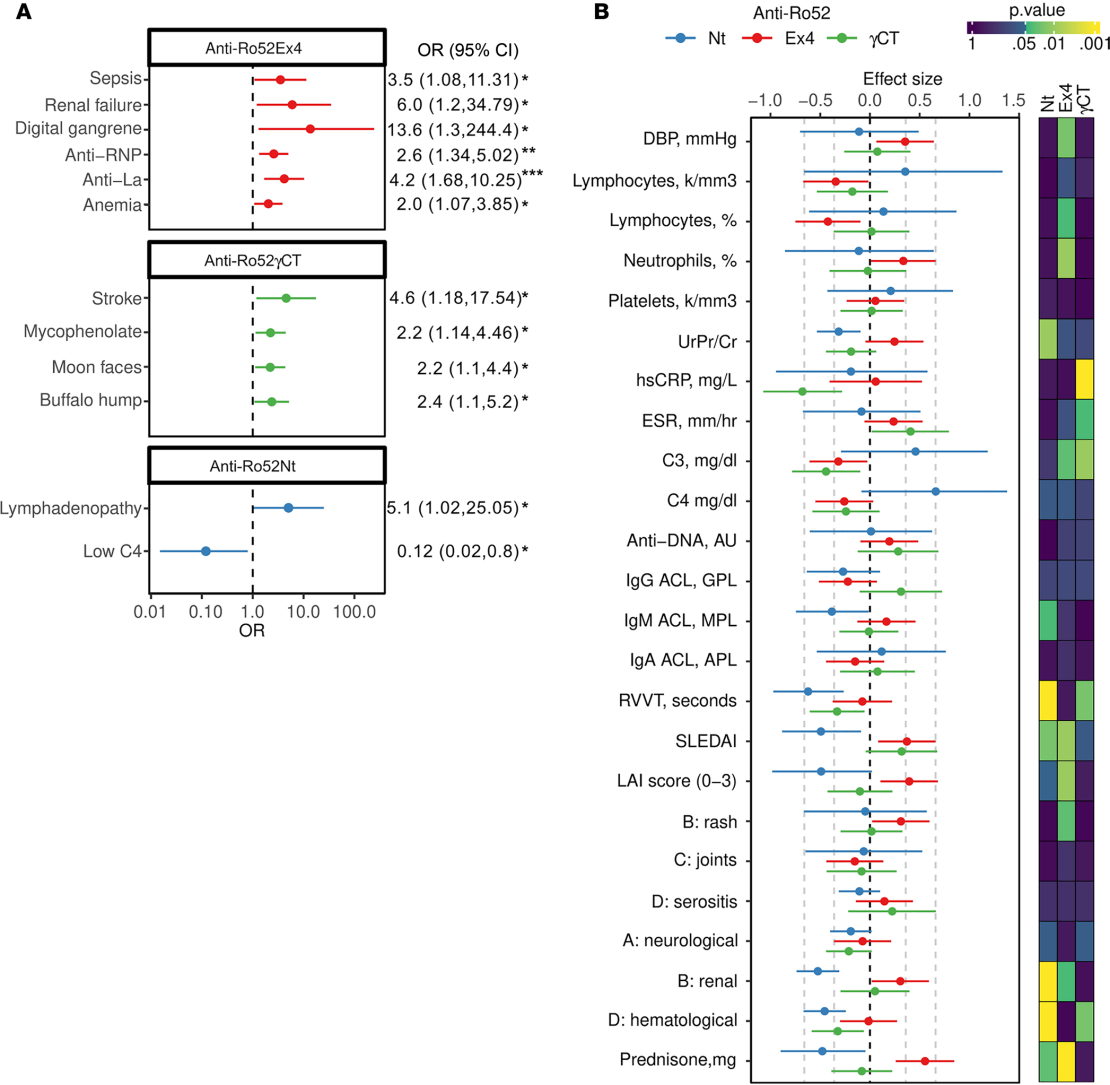
Anti-Ro52 antibody subsets are associated with distinct clinical endotypes in SLE. (**A**) Clinical and laboratory associations present during the clinical course of SLE according to anti-Ro52 antibody type. OR were calculated against patients with SLE negative for the corresponding anti-Ro52 autoantibody type using a 2 × 2 table. Error bars correspond to 95% CI. Associations were tested using χ^2^ test or Fisher’s exact test. (**B**) Left panel, effect size (Cohen’s d) between patients with SLE positive and negative for each anti-Ro52 antibody type over clinical variables and disease activity evaluated at time of visit. Right panel, heatmap of –log_10_ (*P* values) showing the significant associations among anti-Ro52 autoantibody types and variables obtained at time of visit. *P* values were calculated with the Student’s *t* test. DBP, diastolic blood pressure; UrPr/Cr, urinary protein/creatinine ratio; hsCRP, high-sensitivity c-reactive protein; ESR, erythrocyte sedimentation rate; ACL, anti-cardiolipin antibodies; RVVT, dilute russell viper venom time; SLEDAI, Systemic Lupus Erythematosus Disease Activity Index; LAI, Lupus Activity Index. *P < 0.05, **P < 0.01, *** *P <* 0.001.

From the analysis of Ro52 expression in SLE neutrophils and further mapping of antibody subsets based on recognition of Ro52 isoforms, antibodies to Ro52 in SLE are classified in 3 subsets: anti-Ro52Nt, anti-Ro52Ex4, and anti-Ro52γCt antibodies. Interestingly, the distinct antibodies to Ro52 identify clinically relevant endotypes within SLE (Figure 6). Anti-Ro52Nt antibody positivity was associated with lymphadenopathy, and intriguingly, these antibodies were less likely to be associated with lower levels of the complement protein C4 (Figure 6A and Supplemental Table 2). Antibodies to Ro52Ex4 were significantly associated with history of sepsis, renal failure, digital gangrene, anemia, and antibodies to La and RNP (Figure 6A and Supplemental Table 2). In contrast, patients with anti-Ro52γCT antibodies showed an increased frequency of stroke and features of secondary Cushing’s syndrome (i.e., moon faces and buffalo hump), and they were more likely treated with mycophenolate (Figure 6A and Supplemental Table 2).

At time of visit, the presence of anti-Ro52Nt antibodies showed limited value for SLE disease activity. Rather, these antibodies showed negative associations with laboratory and clinical features linked to disease activity. For instance, this group of patients showed lower Systemic Lupus Erythematosus Disease Activity Index (SLEDAI), lupus activity index (LAI), and renal and hematologic activity; higher C3 and C4; and low use of prednisone (Figure 6B and Supplemental Table 3). In contrast, anti-Ro52Ex4 antibodies were associated with lower lymphocyte and higher neutrophil counts, lower C3, higher SLEDAI and LAI, increased rash and renal activity, and higher prednisone doses (Figure 6B and Supplemental Table 3). Moreover, anti-Ro52γCT antibodies were associated with elevated ESR, lower high-sensitivity C-reactive protein (hsCRP) and C3, and a tendency for higher SLEDAI score (Figure 6B and Supplemental Table 3).

Despite the prominent overlap with antibodies to Ro52Ex4, anti-Ro52‘classic’ antibodies were only associated with anemia, anti-La, and sepsis but not with features of disease activity as anti-Ro52Ex4 antibodies (Supplemental Tables 2 and 3). These differences are likely explained because anti-Ro52Nt antibodies, which negatively associate with disease activity, are within the pool of anti-Ro52‘classic’ antibodies. Moreover, anti-Ro52Ex4 antibody positivity was found in 26 additional patients negative for anti-Ro52‘classic’ antibodies. Thus, autoantibodies targeting specific domains in Ro52 variants appear to be more informative than anti-Ro52‘classic’ antibodies to identify clinical subsets in SLE.

### Anti-Ro52 antibody subsets exhibit distinct transcriptional profiles in SLE.

**Figure 7:**
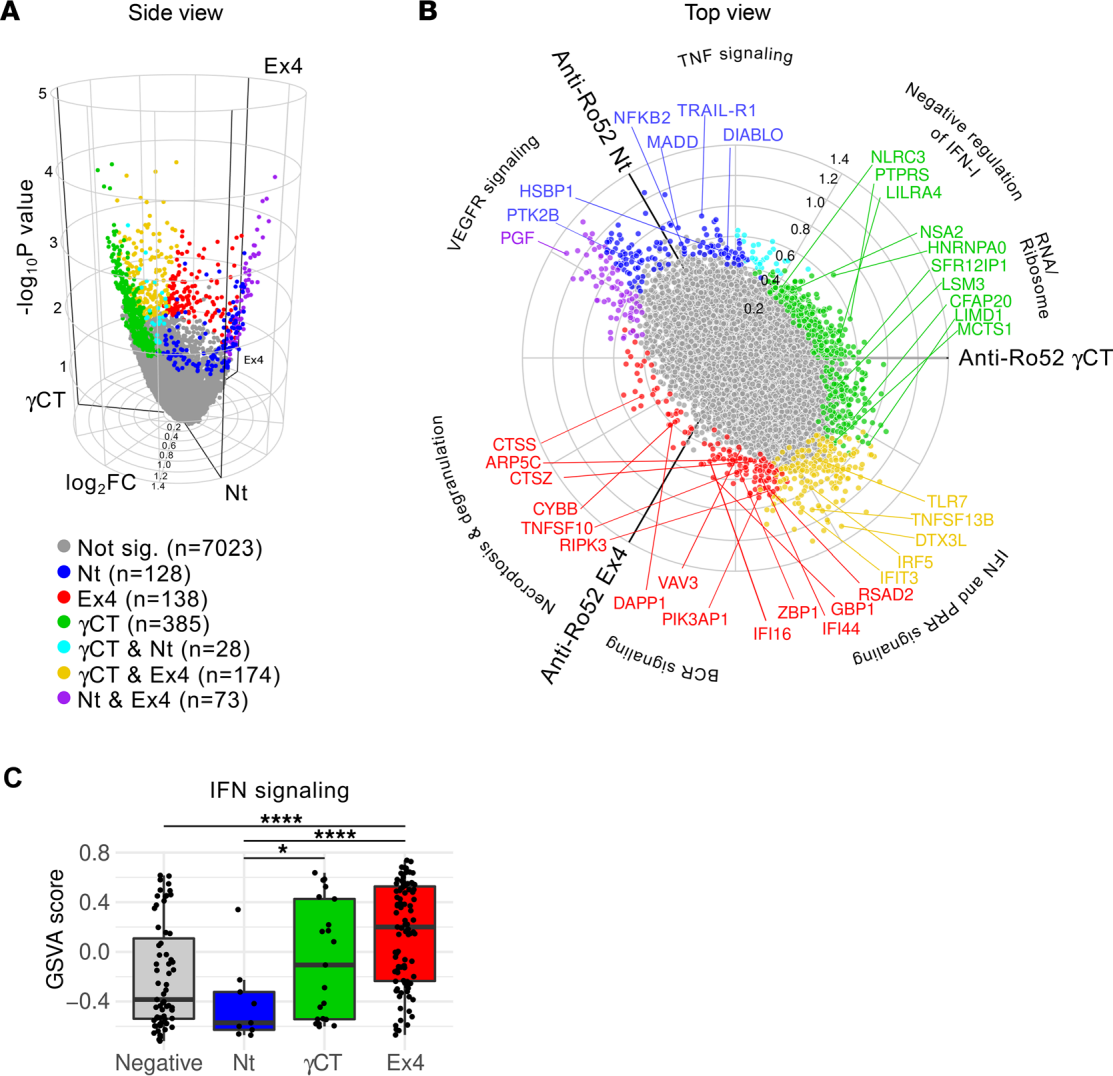
Anti-Ro52 antibody subsets are associated with distinct transcriptional immune-mediated pathways in SLE. (**A** and **B**) Three-way differentially expressed transcript (DET) analysis between patients with SLE positive for anti-Ro52Ex4 (*n =* 95), anti-Ro52Nt (*n =* 9), and anti-Ro52γCT (*n =* 21) antibodies. DET between the 3 anti-Ro52 antibody types (*n =* 926) are shown in a 3D volcano plot (**A**) and in a radial plot (**B**). Significance was calculated using the volcano3D package by combining the results of the *F* test and pairwise comparisons between anti-Ro52Ex4, anti-Ro52Nt, and anti-Ro52γCT, using a multivariate linear model adjusted by anti-DNA positivity and SLEDAI. Color code denotes significant DET in anti-Ro52Ex4 (red), anti-Ro52γCT (green), anti-Ro52Nt (blue), and overlapping genes between anti-Ro52Ex4 and anti-Ro52γCT (yellow), anti-Ro52Nt and anti-Ro52Ex4 (purple), and anti-Ro52γCT and anti-Ro52Nt (light blue). Representative genes from the enriched pathways on each DET subset are labeled. (**C**) Activity of the IFN pathway in patients negative for anti-Ro52 (*n =* 63) and patients with SLE positive for anti-Ro52Ex4 (*n =* 95), anti-Ro52Nt (*n =* 9), and anti-Ro52γCT (*n =* 21) antibodies. Pathway activity was calculated using gene set variation (GSVA) score. Comparisons between groups were done using the pairwise Wilcoxon test. *P < 0.05, ****P < 0.0001.

Patients with SLE display unique blood transcriptional signatures associated with immune pathways activated during active disease (29). In particular, it is interesting that the IFN signature has been linked with antibodies to the Ro particle (30). Since anti-Ro52 antibody subsets correlated with distinct clinical features in SLE, we further addressed the relationship of these antibodies with transcriptional fingerprints activated in SLE. Using whole blood gene expression data collected in parallel with the samples used to measure anti-Ro52 antibodies, we performed a 3-way comparison between patients with SLE positive for antibodies to Ro52Nt, Ro52Ex4, and Ro52γCt (Figure 7A).

We identified 926 differentially expressed transcripts (DETs) between the 3 anti-Ro52 antibodies (Figure 7A and Supplemental File 1). DETs associated with anti-Ro52Ex4 (*n =* 138) were predominantly enriched in genes involved in immune mediated pathways including IFN, intracellular DNA sensing, B cell receptor signaling, necroptosis, and degranulation (Figure 7B, Supplemental File 1, and Supplemental Figure 3). Interestingly, a group of common DETs between anti-Ro52γCT and anti-Ro52Ex4 (*n =* 174) was mainly enriched in pathways related to IFN signaling (including increased expression of TNFSF13B, TLR7, IFIT3, and IRF5) and antigen presentation. In contrast, DETs exclusive to anti-Ro52γCT (*n =* 385) contained genes related to p53 signaling, RNA metabolism, ribosome biogenesis, mitochondrial function, and negative regulation of IFN-I (Figure 7B, Supplemental File 1, and Supplemental Figure 3). DETs associated with anti-Ro52Nt antibodies lacked enrichment of IFN-stimulated genes (ISGs), which is consistent with the low disease activity in this small group of patients. Instead, these antibodies were associated with increased vascular endothelial growth factor, TNF signaling, and apoptosis (Figure 7B, Supplemental File 1, and Supplemental Figure 3). Using gene set variation analysis (GSVA) (31) to quantify the activity of selected pathways on individual patients with SLE, we further confirmed that antibodies to Ro52Ex4 have the most significant association with the IFN signature, followed by anti-Ro52γCT antibodies, and no association of anti-Ro52Nt antibodies with the IFN signature was found (Figure 7C).

## Discussion

The origin and mechanisms underlying the production of autoantigens in SLE remain unclear. Since core autoantigens in SLE — such as Ro52, Ro60, La, histones, dsDNA, and RNPs — are normally expressed under steady-state conditions, it has been hypothesized that self-immunogenic forms of these molecules are generated under unique inflammatory environments amplified in SLE. When we searched for autoantigens targeted by SLE serum in in vivo IFN-activated neutrophils from patients with SLE, we were struck by the finding that Ro52 is a prominent IFN-induced autoantigen in SLE neutrophils that is absent or minimally detected at the protein level in both healthy control and steady-state SLE neutrophils. Moreover, IFN-activated SLE neutrophils contained multiple Ro52 species recognized by autoantibodies that were absent in PBMCs. By combining the analysis of an RNA-Seq data set of peripheral blood SLE leukocytes, epitope mapping with commercial anti-Ro52 antibodies, and the study of a large cohort of patients with SLE, we further concluded that the large majority of antibodies to Ro52 in SLE are directed against an epitope encoded by exon 4 in *TRIM21* — found in Ro52α and Ro52γ — which is the major target of anti-Ro52 antibodies in in vivo IFN-activated SLE neutrophils. The significant association of anti-Ro52Ex4 and anti-Ro52γCT antibodies with the IFN signature provides additional evidence that this set of autoantibodies is mechanistically related to the IFN-induced activation in SLE.

Although the expression of Ro52α and Ro52γ can explain the binding of anti-Ro52Ex4 antibodies to SLE neutrophils, the sole expression of these isoforms (molecular weights 52 and ~33 kDa, respectively) is insufficient to elucidate the origin of the broad range of bands containing the Ro52Ex4 epitope, which are detected in IFN-activated SLE neutrophils. While we cannot discard the existence of additional Ro52 isoforms, an alternative hypothesis is that Ro52α and Ro52γ suffer additional modifications, creating complex patterns of Ro52 detection. In the case of Ro52α, cleavage, and/or trimming of the N-terminal and C-terminal regions, leaving fragments containing the core Ro52Ex4 sequence intact could explain the detection of multiple Ro52 species below 52 kDa. Regarding Ro52γ, this isoform would require modifications that increase the molecular weight of the protein. It is noteworthy that both SLE and healthy control neutrophils contain molecular weight bands below 33 kDa, and they were detected by the 3 commercial antibodies against Ro52Ex4, supporting that these bands are likely generated from Ro52α/Ro52γ. As an additional hypothesis, it is possible that Ro52 is normally degraded in neutrophils under steady-state conditions but accumulates during SLE disease activity as a result of increased expression and likely less degradation. Although neither of these possibilities are exclusive of each other, the most striking finding is that the protein species containing Ro52Ex4 are both enriched in in vivo IFN-activated neutrophils and are the main target of anti-Ro52 antibodies in SLE. This finding is unlikely to be coincidental but is rather mechanistically related.

Ro52α is an abundant isoform constitutively expressed by several cell types, such as keratinocytes and immune cells (2, 9, 14). If Ro52α is the main immunogen in SLE, it is intriguing that the major target of anti-Ro52 antibodies is limited to Ro52Ex4, sparing the C-terminal half of the molecule (28). In contrast, however, Ro52γ is targeted both at Ro52Ex4 and the C-terminal domain, offering it an alternative self-antigen to explain the subsets of anti-Ro52 antibodies found in SLE — i.e., anti-Ro52Nt, anti-Ro52Ex4, and anti-Ro52γCT — as well as the limited humoral response against C-terminal Ro52α. The proposal that the N-terminal and C-terminal regions of Ro52α may be cleaved/trimmed, enriching for immunogenic fragments containing Ro52Ex4, is also a potential mechanism to explain the antibody specificity to Ro52Ex4 without targeting the C-terminal domain. Indeed, both models are not exclusive; they are complementary. Importantly, whereas it is possible that other cell types in different tissues may generate similar Ro52 patterns as IFN-activated neutrophils in SLE, our work demonstrates that neutrophils are the main cellular source of multiple Ro52 protein species containing Ro52Ex4 in peripheral blood in SLE. In this scenario, large amounts of immunogenic Ro52 may become accessible to the immune system as a result of neutrophil death in SLE, which may initiate an immune response to Ro52.

Interestingly, the striking difference in the phenotype of 2 *Ro52/Trim21*-KO mice, in which one develops lupus-like disease and the other has a normal life span (2, 9), has been attributed to the potential production of a truncated Ro52 protein that is overexpressed in the mouse that develops lupus (32, 33). Like Ro52γ, except for the lack of the unique Ro52γ-CT domain, the predicted truncated protein carries the RING domain, B-box, and coiled-coil domain containing the homologous sequence of Ro52Ex4, which is instead encoded by exon 5 in mouse *Trim21* (32). In the context of our findings, it is possible that lupus-like disease in these mice is driven by the truncated Ro52γ-like protein; this may work either as an autoantigen or through a unique proinflammatory function resulting from the lack of the B30.2/PRYSPRY domain, which mediates protein-to-protein interactions and Fc receptor function (34, 35).

Our finding that SLE neutrophils activated by IFN are highly enriched in Ro52 species targeted by the majority of anti-Ro52 antibodies in SLE underscores neutrophils as the main cellular sources of self-immunogenic Ro52 in peripheral blood in SLE. Moreover, the study uncovers the isoform-specific domains Ro52Ex4 and Ro52γCT as the core targets of anti-Ro52 antibodies in SLE that could be used as potential biomarkers of disease state and to unravel disease mechanisms associated with SLE.

## Methods

### Study design and participants.

The objective of the study was to identify neutrophil autoantigens with unique patterns of expression linked to in vivo activation by IFN in SLE. PBMCs and peripheral blood neutrophils were purified from 19 consecutive patients with SLE. Briefly, after Ficoll-Hypaque isolation of mononuclear cells, neutrophils were isolated by 2 cycles of RBC lysis using ACK lysing buffer (Quality Biological). Cells were lysed and boiled immediately after purification in SDS-sample buffer. Sera from 80 healthy controls and 191 patients with SLE from the SPARE (26, 27) cohort were screened for the presence of anti-Ro52 antibodies. SPARE is a prospective observational cohort that has been extensively described previously (26). Briefly, adult patients (age 18–75 years old) who met the definition of SLE per the revised American College of Rheumatology classification criteria were eligible into the study (36). At baseline, the patient’s medical history was reviewed, and information on current medications was recorded. Patients were followed-up over a 2-year period. Patients were treated according to standard clinical practice. Disease activity was assessed using the Safety of Estrogens in Lupus Erythematosus: National Assessment (SELENA) version of the SLEDAI (37) and physician global assessment (PGA) (38). C3, C4, anti-dsDNA (Crithidia), complete blood cell count, and urinalysis were performed at every visit. Study participants also underwent whole blood gene expression analysis at baseline using the Affymetrix GeneChip HT HG-U133+ (26, 27).

### Autoantigen discovery.

PBMC and neutrophil lysates from patients with SLE were used to detect IFIT3 by immunoblotting as a marker of IFN activation (21). To identify neutrophil-specific SLE autoantigens linked to IFN activation, cell lysates with low and high IFIT3 expression were pooled according to cell type and IFIT3 expression and were screened by immunoblotting sera from 20 consecutive patients with SLE. The identity of autoantigens of interest was further determined by 2-dimensional electrophoresis and MS, as previously described (39).

### Antibodies.

Mouse monoclonal anti–human IFIT3 (H00003437-B01) was purchased from Abnova, mouse monoclonal anti–human Ro52 (clone D-12) was purchased from Santa Cruz Biotechnology Inc., rabbit anti–N-terminal TRIM21 polyclonal antibody was purchased from Origene (TA335782), rabbit anti–human TRIM21 polyclonal antibody was purchased from Proteintech (121081-1-AP), mouse anti–human TRIM21 monoclonal antibody was purchased from Proteintech (671361-1-Ig), rabbit anti–C-terminal TRIM21 polyclonal antibody was purchased from MilliporeSigma (AV381248), mouse anti–human β-actin was purchased from MilliporeSigma (A5316), and mouse anti–human histone H3 was purchased from Cell Signaling Technology (96C10).

### Cloning, production, and expression of recombinant Ro52 isoforms and domains.

The coding sequence of Ro52α was amplified from SLE neutrophil cDNA and was used as a template to generate Ro52β and Ro52γ by deleting exons 4 and 6, respectively. Exon deletion was performed using the Q5 Site-Directed Mutagenesis Kit (New England Biolabs). All Ro52 isoforms were cloned into pcDNA3.1 and pET28a(+). The sequences spanning exons 4, 4 and 5, 3 and 4, and 3–5 were amplified by PCR using Ro52α as template and cloned into pET28a(+). Recombinant proteins were expressed in *E*. *coli* BL21 (DE3) and purified by Ni-NTA affinity chromatography. All recombinant proteins are N-terminal His tagged. The complete C-terminal domain of Ro52γ (Ro52γCT) was generated as a 35 mer synthetic peptide (SPHHSGSRHSQSVADTFRRSETSEAWRHPAEHTWK). HEK-293T cells were transiently transfected with plasmids to express Ro52 isoforms using lipofectamine 2000 (Invitrogen). HEK-293 cells were lysed 48 hours after transfection and used for immunoblotting analyses.

### Detection of antibodies to Ro52, Ro52Ex4, and Ro52γCT.

“Classical” anti-Ro52 antibodies were detected by ELISA using a commercial kit from INOVA (QUANTA Lite SS-A 52, no. 704505). Anti-Ro52Ex4 and anti-Ro52γCT antibodies were measured in serum/plasma by homemade ELISA. Briefly, Nunc MaxiSorp plates were coated with 4 μg/mL recombinant Ro52 exons 3 and 4 to detect anti-Ro52Ex4 antibodies, and 5 μg/mL of Ro52γCT were covalently attached to Nunc CovaLink NH plates to detect anti-Ro52γCT antibodies. The plates were blocked for 1 hour with phosphate buffered saline plus 0.1% Tween-20 (PBST) with 3% nonfat milk. Serum/plasma was diluted 1:1,000 in PBST 1% nonfat milk and assayed in duplicate using antigen-conjugated plates and plates without antigen for background subtraction. HRP-conjugated goat anti–human IgG was used as a secondary antibody (diluted at 1:10,000 in PBST 1% nonfat milk). Anti-Ro52Ex4 and anti-Ro52γCT antibody arbitrary units were calculated using a standard curve made of a serial diluted serum from a high-titer SLE patient. Anti-Ro52Nt antibodies were detected by immunoblotting using recombinant Ro52α, Ro52β, Ro52γ, and the Ro52 sequence encoded by exons 3 and 4.

### Differential gene expression and enrichment analyses.

Gene expression analysis from the SPARE cohort was previously described (27). CEL files were subjected to RMA background correction and quantile normalization using the Oligo package (29). To select only expressed genes in whole blood, we filtered out transcripts that had a raw signal < 100 in less than 10% of samples with the genefilter R package. All calculations and analyses were performed using R (ver 4.2.1) and Bioconductor (ver 3.15.2) (40). DETs were analyzed using the R package ‘limma’ using a multivariate linear model adjusted of anti-dsDNA positivity and SLEDAI (41). Gene set enrichment analyses were done using the online platform Metascape.org (42). Three-way differential gene expression analyses were done using the package ‘volcano3D’ (43) by combining the results from the pairwise comparisons between anti-Ro52Ex4, anti-Ro52γCT, and anti-Ro52Nt antibodies, along the *F* test calculated with the R package ‘limma’. To annotate the transcriptomes associated to each anti-Ro52 antibody type, we performed gene set enrichment analyses using the online platform Metascape.org (42). Briefly, in order to do the enrichment analyses, we split the DET into lists according to the results of 3-way analysis. Then, the lists of genes were uploaded into the Metascape.org platform. Adjusted *P* < 0.05 was considered as significant. Activity of the IFN pathways was calculates using GSVA with the R package GSVA (31).

### Isoform analysis in RNA-Seq data.

To discover new isoforms of the Ro52 antigen, we reanalyzed RNA-Seq data from neutrophils, cMo, and T cells from 24 patients with SLE and 12 healthy controls from the publicly available data set GSE149050 (23). In addition, we analyzed RNA-Seq data from keratinocytes from 7 HC and 7 SLE subjects deposited at GSE123949 (24). Briefly, the fastq files were aligned to the human genome reference build 38 (GRCh38/hg38) using the splicing-aware aligner HISAT2 (44). Sample’s BAM files were further processed using StringTie to quantify de novo–assembled transcripts (45). Visualization of the de novo assembled Ro52 (TRIM21) transcripts was done using Ballgown (46).

### Statistics.

Comparisons of continuous variables between 2 groups were done using 2-tailed Student’s *t* test. Fisher’s exact test was used for univariate analysis on SPARE cohort variables, the exact 2 × 2 package in R was used to calculate the *P* value, OR, and 95% CI. Effect size (Cohen’s d) between anti-Ro52 positive and negative SLE subjects was calculated using the ‘psych’ R package (47). Pairwise comparisons between pathway activity were done using Wilcoxon’s pairwise test. Statistical significance was set at *P <* 0.05. The statistical analyses were carried with the R software version 4.2.1.

### Study approval.

All samples were obtained under informed written consent approved by the Johns Hopkins IRB.

## Author contributions

All authors were involved in drafting the article or revising it critically for important intellectual content, and all authors approved the final version to be published. Study conception and design were contributed by FA. Experimental work was contributed by EGB, MJW, MP, BA, AIC, VA, DPF, ED, and FA. Acquisition of data was contributed by EGB, MJW, JL, MP, BA, AIC, VA, DPF, DWG, MP, and FA. Analysis and interpretation of data were contributed by EGB, MJW, JL, BA, DG, ED, MP, and FA.

## Supplementary Material

Supplemental data

Supplemental data set 1

## Version 1 08/30/2022

In-Press Preview

## Version 2 10/10/2022

Electronic publication

## Version 3 10/12/2022

The text was updated to remove the word "potentially" in three places
