# Supplementary material for: Alternative exon usage in TRIM21 determines the antigenicity of Ro52/TRIM21 in systemic lupus erythematosus
Source: JCI Insight. 2022 Oct 10;7(19):e163795. doi: 10.1172/jci.insight.163795 (PMC9675474; doi:10.1172/jci.insight.163795)
Supplement: Supplemental data [file jciinsight-7-163795-s264.pdf]

Supplemental materials for

Alternative exon usage in *TRIM21* determines the antigenicity of Ro52/TRIM21 in systemic lupus erythematosus

Authors

Eduardo Gomez-Bañuelos, M. Javad Wahadat, Jessica Li, Merlin Paz, Brendan Antiochos, Alessandra Ida Celia, Victoria Andrade, Dylan P. Ferris, Daniel Goldman, Erika Darrah, Michelle Petri, Felipe Andrade*

*Corresponding author. Email: andrade@jhmi.edu

The file includes:

Supplemental Figure 1. Two-dimensional mapping of autoantigens overexpressed in SLE neutrophils.

Supplemental Figure 2. TRIM21/Ro52 splicing variants in SLE keratinocytes.

Supplemental Figure 3. Heatmap of enriched gene ontology (GO) terms across differentially expressed transcripts associated with anti-Ro52 antibody types.

Supplemental Table 1. Demographic characteristics of SLE patients from SPARE.

Supplemental Table 2. Clinical and laboratory associations present during the clinical course of SLE according to anti-Ro52 antibody type.

Supplemental Table 3. Clinical, laboratory and disease activity associations at time of visit according to anti-Ro52 antibody type.

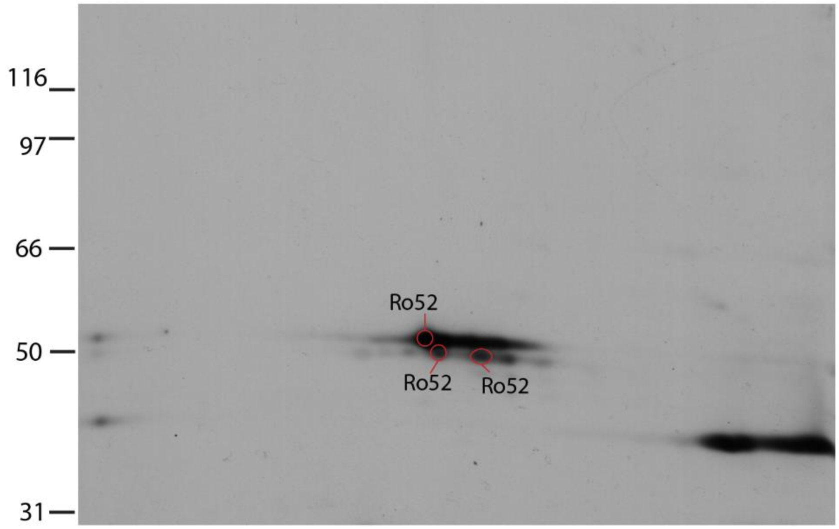

Supplemental Figure 1. Two-dimensional mapping of autoantigens overexpressed in SLE neutrophils. Cell lysates from IFN-high SLE neutrophils were resolved by two-dimensional electrophoresis and immunoblotted using SLE patient sera that detected the same pattern of bands from Figure 1C. Once the antigens of interest were mapped, three spots were sliced from a two-dimensional gel stained with GelCode blue (Thermo Scientific) and analyzed by mass spectrometry (Proteomics Core/Mass Spectrometry Facility, Johns Hopkins School of Medicine). The proteins were identified as Ro52/TRIM21.

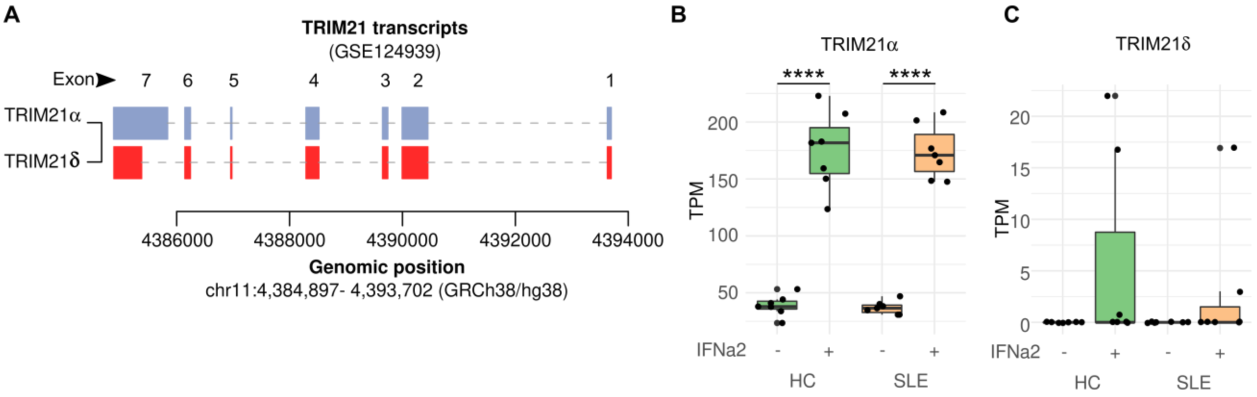

Supplemental Figure 2. TRIM21/Ro52 splicing variants in SLE keratinocytes. (**A**) Schematic representation of the transcripts corresponding to TRIM21 isoforms found in a publicly available RNAseq dataset (GSE124939) of keratinocytes from SLE patients (n=7) and healthy controls (HC, n=7) using the 'new tuxedo' pipeline. Each solid block represents an exon. (**B** and **C**) Differential expression analyses of TRIM21α (**B**) and TRIM21δ (**C**) in HC and SLE keratinocytes according to IFNa2 stimulation. Pairwise comparisons between HC and SLE were done using Wilcoxon's Test. ****p<0.0001.

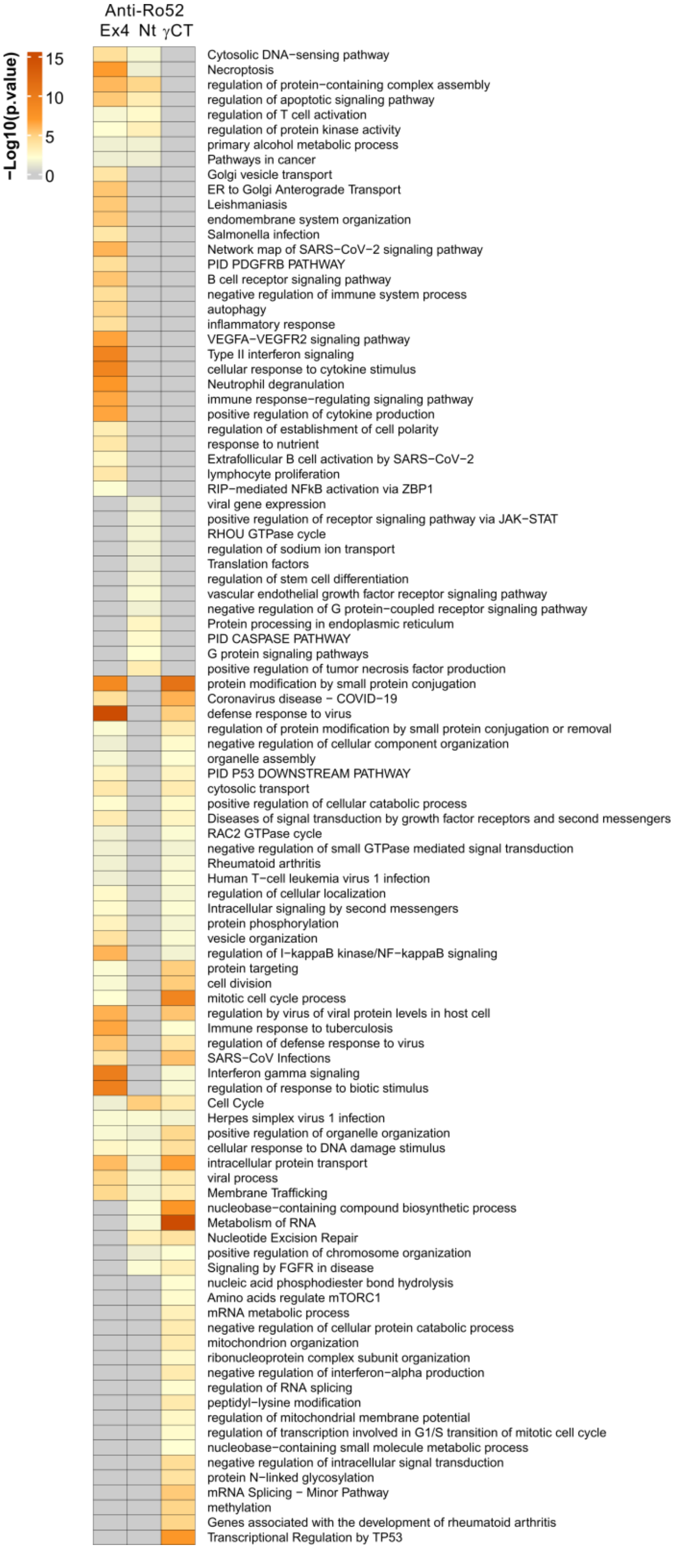

Supplemental Figure 3. Heatmap of enriched gene ontology (GO) terms across differentially expressed transcripts associated with anti-Ro52 autoantibody types. The top 100 enriched clusters color by p-value associated with anti-Ro52Ex4, anti-Ro52γCT, and anti-Ro52Nt antibodies are shown. Enrichment analyses were carried out using the multiple-list option from Metascape.org.

Supplemental Table 1. Demographic characteristics of SLE patients from SPARE

Variable	n	n (%)
Female Sex	190	177 (93%)
Race	190	
White		100 (53%)
Black		74 (39%)
Asian		9 (4.7%)
Other		7 (3.7%)
Smoking	190	15 (7.9%)
SLEDAI	187	2 (0, 15)[1]
Renal SLE	190	99 (52%)
Sjogren's Syndrome	190	48 (25%)
Anti-DNA	190	123 (65%)
Anti-Sm	189	38 (20%)
Anti-Ro52	190	75 (39%)
Anti-La	189	28 (15%)
Anti-RNP	189	52 (28%)
Current treatment		
Prednisone	190	68 (36%)
Hydroxicholoroquine	190	167 (88%)
Cytotoxic	190	117 (62%)

[1]Median (min-max). Cytotoxic treatment includes: Cyclophosphamide, Mycophenolic acid, Azathioprine, and Methotrexate. Demographic, clinical or laboratory data were only available for 187-190/191 SLE patients.

Supplemental Table 2. Clinical and laboratory associations present during the clinical course of SLE according to anti-Ro52 antibody type.

	Anti-Ro52											
	'classic'			Nt			Ex4			γCT		
Clinical	Negative N=115	Positive n= 75	p.value	Negative n = 181	Positive n = 9	p.value	Negative n = 96	Positive n = 94	p.value	Negative n = 146	Positive n = 44	p.value
Female	108(93.9)	69(92)	0.77	168(92.8)	9(100)	1	89(94.7)	88(91.7)	0.57	135(92.5)	42(95.5)	0.74
Black	48(41.7)	26(34.7)	0.36	71(39.2)	3(33.3)	1	36(38.3)	38(39.6)	0.88	54(37)	20(45.5)	0.38
Past smoker	43(37.4)	27(36)	0.88	65(35.9)	5(55.6)	0.29	30(31.9)	40(41.7)	0.18	55(37.7)	15(34.1)	0.72
Fever	41(35.7)	20(26.7)	0.21	57(31.5)	4(44.4)	0.47	32(34)	29(30.2)	0.64	45(30.8)	16(36.4)	0.58
**Lymphadenopathy**	44(38.3)	37(49.3)	0.14	**74(40.9)**	**7(77.8)**	**0.04**	39(41.5)	42(43.8)	0.77	62(42.5)	19(43.2)	1
Malar rash	59(51.3)	43(57.3)	0.46	98(54.1)	4(44.4)	0.74	48(51.1)	54(56.2)	0.56	78(53.4)	24(54.5)	1
Discoid	24(20.9)	17(22.7)	0.86	38(21)	3(33.3)	0.41	22(23.4)	19(19.8)	0.6	31(21.2)	10(22.7)	0.84
Photosensitivity	65(56.5)	37(49.3)	0.37	97(53.6)	5(55.6)	1	51(54.3)	51(53.1)	0.88	75(51.4)	27(61.4)	0.3
Mouth ulcers	69(60)	40(53.3)	0.37	101(55.8)	8(88.9)	0.08	59(62.8)	50(52.1)	0.14	80(54.8)	29(65.9)	0.22
Alopecia	77(67)	44(58.7)	0.28	117(64.6)	4(44.4)	0.29	61(64.9)	60(62.5)	0.76	88(60.3)	33(75)	0.11
Raynaud	65(56.5)	42(56)	1	102(56.4)	5(55.6)	1	54(57.4)	53(55.2)	0.77	87(59.6)	20(45.5)	0.12
SCLE	4(3.5)	7(9.3)	0.12	10(5.5)	1(11.1)	0.42	5(5.3)	6(6.2)	1	8(5.5)	3(6.8)	0.72
Bullous	1(0.9)	0(0)	1	1(0.6)	0(0)	1	1(1.1)	0(0)	0.5	1(0.7)	0(0)	1
Vasculitis	15(13)	12(16)	0.67	25(13.8)	2(22.2)	0.62	12(12.8)	15(15.6)	0.68	22(15.1)	5(11.4)	0.63
Leg ulcers	2(1.7)	2(2.7)	0.65	3(1.7)	1(11.1)	0.18	3(3.2)	1(1)	0.37	4(2.7)	0(0)	0.58
Panniculitis	2(1.7)	5(6.7)	0.12	6(3.3)	1(11.1)	0.29	3(3.2)	4(4.2)	1	4(2.7)	3(6.8)	0.2
Livedo	43(37.4)	21(28)	0.21	60(33.1)	4(44.4)	0.49	35(37.2)	29(30.2)	0.36	53(36.3)	11(25)	0.2
Arthralgias	109(94.8)	70(93.3)	0.76	171(94.5)	8(88.9)	0.42	90(95.7)	89(92.7)	0.54	137(93.8)	42(95.5)	1
Arthritis	92(80)	54(72)	0.22	140(77.3)	6(66.7)	0.44	75(79.8)	71(74)	0.39	110(75.3)	36(81.8)	0.42
Erosions	1(1.3)	1(2.1)	1	2(1.7)	0(0)	1	1(1.5)	1(1.8)	1	2(2.2)	0(0)	1
Myositis	8(7)	8(10.7)	0.43	14(7.7)	2(22.2)	0.17	8(8.5)	8(8.3)	1	12(8.2)	4(9.1)	0.77
Pleuritis	61(53)	34(45.3)	0.37	91(50.3)	4(44.4)	1	52(55.3)	43(44.8)	0.19	71(48.6)	24(54.5)	0.61
Pericarditis	32(28.1)	16(21.3)	0.31	47(26.1)	1(11.1)	0.45	26(28)	22(22.9)	0.5	35(24.1)	13(29.5)	0.55
Renal SLE	60(52.2)	39(52)	1	95(52.5)	4(44.4)	0.74	43(45.7)	56(58.3)	0.11	79(54.1)	20(45.5)	0.39
Proteinuria	58(50.4)	35(46.7)	0.66	91(50.3)	2(22.2)	0.17	40(42.6)	53(55.2)	0.08	73(50)	20(45.5)	0.61
Hematuria	35(30.4)	27(36)	0.43	59(32.6)	3(33.3)	1	26(27.7)	36(37.5)	0.17	44(30.1)	18(40.9)	0.2
**Renal failure**	5(4.3)	4(5.3)	0.74	9(5)	0(0)	1	**1(1.1)**	**8(8.3)**	**0.03**	7(4.8)	2(4.5)	1
Seizure	8(7)	4(5.3)	0.77	11(6.1)	1(11.1)	0.45	8(8.5)	4(4.2)	0.25	10(6.8)	2(4.5)	0.74
Psychosis	2(1.7)	2(2.7)	0.65	3(1.7)	1(11.1)	0.18	2(2.1)	2(2.1)	1	4(2.7)	0(0)	0.58
OBS	6(5.2)	3(4)	1	8(4.4)	1(11.1)	0.36	7(7.4)	2(2.1)	0.1	6(4.1)	3(6.8)	0.44
Aseptic meningitis	1(0.9)	1(1.3)	1	1(0.6)	1(11.1)	0.09	2(2.1)	0(0)	0.24	1(0.7)	1(2.3)	0.41
**Stroke**	5(4.3)	2(2.7)	0.71	6(3.3)	1(11.1)	0.29	6(6.4)	1(1)	0.06	**3(2.1)**	**4(9.1)**	**0.05**
Depression	51(44.3)	23(30.7)	0.07	70(38.7)	4(44.4)	0.74	41(43.6)	33(34.4)	0.23	61(41.8)	13(29.5)	0.16
Lupus Headache	10(8.7)	6(8)	1	14(7.7)	2(22.2)	0.17	11(11.7)	5(5.2)	0.12	14(9.6)	2(4.5)	0.37
MM	1(0.9)	1(1.3)	1	2(1.1)	0(0)	1	1(1.1)	1(1)	1	1(0.7)	1(2.3)	0.41
Cognitive imp.	10(8.7)	5(6.7)	0.78	15(8.3)	0(0)	1	5(5.3)	10(10.4)	0.28	12(8.2)	3(6.8)	1
Optic neuritis	0(0)	0(0)	1	0(0)	0(0)	1	0(0)	0(0)	1	0(0)	0(0)	1
Cranial neuritis	3(2.6)	4(5.3)	0.44	6(3.3)	1(11.1)	0.29	4(4.3)	3(3.1)	0.72	6(4.1)	1(2.3)	1
Peripheral neuropathy	4(3.5)	5(6.7)	0.32	8(4.4)	1(11.1)	0.36	4(4.3)	5(5.2)	1	6(4.1)	3(6.8)	0.44
Transverse myelitis	3(2.6)	1(1.3)	1	4(2.2)	0(0)	1	2(2.1)	2(2.1)	1	2(1.4)	2(4.5)	0.23
**Anemia**	**75(65.2)**	**62(82.7)**	**0.01**	130(71.8)	7(77.8)	1	**61(64.9)**	**76(79.2)**	**0.04**	108(74)	29(65.9)	0.34
Hemolysis	9(7.8)	6(8)	1	15(8.3)	0(0)	1	8(8.5)	7(7.3)	0.79	9(6.2)	6(13.6)	0.12
Coombs	19(16.5)	15(20)	0.57	34(18.8)	0(0)	0.37	14(14.9)	20(20.8)	0.34	25(17.1)	9(20.5)	0.66
Leukopenia	55(47.8)	31(41.3)	0.46	83(45.9)	3(33.3)	0.52	42(44.7)	44(45.8)	0.88	69(47.3)	17(38.6)	0.39
Lymphopenia	50(43.5)	32(42.7)	1	78(43.1)	4(44.4)	1	44(46.8)	38(39.6)	0.38	67(45.9)	15(34.1)	0.22
Thrombocytopenia	31(27)	18(24)	0.74	47(26)	2(22.2)	1	25(26.6)	24(25)	0.87	37(25.3)	12(27.3)	0.84
Lupus Anticoagulant	6(33.3)	2(16.7)	0.42	7(24.1)	1(100)	0.27	6(35.3)	2(15.4)	0.41	7(25.9)	1(33.3)	1
ACL	72(62.6)	52(69.3)	0.36	119(65.7)	5(55.6)	0.72	57(60.6)	67(69.8)	0.22	91(62.3)	33(75)	0.15
Anti-β2GPI	33(29.2)	25(33.3)	0.63	56(31.3)	2(22.2)	0.72	27(29)	31(32.6)	0.64	44(30.3)	14(32.6)	0.85
SS	30(26.1)	18(24)	0.86	45(24.9)	3(33.3)	0.69	25(26.6)	23(24)	0.74	39(26.7)	9(20.5)	0.44
Myocarditis	2(1.7)	1(1.3)	1	3(1.7)	0(0)	1	1(1.1)	2(2.1)	1	3(2.1)	0(0)	1
Libman-Sacks	1(0.9)	2(2.7)	0.56	3(1.7)	0(0)	1	1(1.1)	2(2.1)	1	3(2.1)	0(0)	1

	Anti-Ro52											
	‘classic’			Nt			Ex4			γCT		
Clinical	Negative N=115	Positive n= 75	p.value	Negative n = 181	Positive n = 9	p.value	Negative n = 96	Positive n = 94	p.value	Negative n = 146	Positive n = 44	p.value
Murmur	58(50.4)	40(53.3)	0.77	94(51.9)	4(44.4)	0.74	50(53.2)	48(50)	0.67	74(50.7)	24(54.5)	0.73
Lung fibrosis	14(12.2)	15(20)	0.15	27(14.9)	2(22.2)	0.63	13(13.8)	16(16.7)	0.69	23(15.8)	6(13.6)	0.82
PHT	13(11.3)	10(13.3)	0.82	22(12.2)	1(11.1)	1	11(11.7)	12(12.5)	1	16(11)	7(15.9)	0.43
Hepatomegaly	3(2.6)	2(2.7)	1	5(2.8)	0(0)	1	3(3.2)	2(2.1)	0.68	4(2.7)	1(2.3)	1
Abnormal LFT	53(46.1)	32(42.7)	0.66	81(44.8)	4(44.4)	1	42(44.7)	43(44.8)	1	66(45.2)	19(43.2)	0.86
Splenomegaly	1(0.9)	4(5.3)	0.08	5(2.8)	0(0)	1	1(1.1)	4(4.2)	0.37	4(2.7)	1(2.3)	1
GI lupus	8(7)	7(9.3)	0.59	14(7.7)	1(11.1)	0.53	7(7.4)	8(8.3)	1	14(9.6)	1(2.3)	0.2
Pancreatitis	3(2.6)	3(4)	0.68	5(2.8)	1(11.1)	0.26	2(2.1)	4(4.2)	0.68	5(3.4)	1(2.3)	1
Anti-DNA	70(60.9)	53(70.7)	0.21	118(65.2)	5(55.6)	0.72	56(59.6)	67(69.8)	0.17	91(62.3)	32(72.7)	0.28
Anti-Sm	19(16.5)	19(25.7)	0.14	37(20.6)	1(11.1)	0.69	15(16)	23(24.2)	0.2	29(20)	9(20.5)	1
**Anti-La**	**7(6.1)**	**21(28.4)**	**< 0.001**	27(15)	1(11.1)	1	**6(6.4)**	**22(23.2)**	**<0.001**	22(15.2)	6(13.6)	1
**Anti-RNP**	27(23.5)	25(33.8)	0.14	51(28.3)	1(11.1)	0.45	**17(18.1)**	**35(36.8)**	**0.01**	42(29)	10(22.7)	0.45
Low CH50	15(13)	13(17.6)	0.41	28(15.6)	0(0)	0.36	12(12.8)	16(16.8)	0.54	20(13.8)	8(18.2)	0.47
Low C3	62(53.9)	48(64)	0.18	106(58.6)	4(44.4)	0.5	50(53.2)	60(62.5)	0.24	80(54.8)	30(68.2)	0.12
**Low C4**	53(46.1)	39(52)	0.46	**91(50.3)**	**1(11.1)**	**0.04**	39(41.5)	53(55.2)	0.06	67(45.9)	25(56.8)	0.23
Obesity	70(60.9)	45(60)	1	108(59.7)	7(77.8)	0.49	60(63.8)	55(57.3)	0.38	90(61.6)	25(56.8)	0.6
**Moon facies**	41(35.7)	30(40)	0.65	68(37.6)	3(33.3)	1	32(34)	39(40.6)	0.37	**48(32.9)**	**23(52.3)**	**0.02**
**Buffalo hump**	21(18.3)	14(18.7)	1	33(18.2)	2(22.2)	0.67	16(17)	19(19.8)	0.71	**22(15.1)**	**13(29.5)**	**0.04**
Truncal obesity	27(23.5)	16(21.3)	0.86	41(22.7)	2(22.2)	1	20(21.3)	23(24)	0.73	28(19.2)	15(34.1)	0.06
Avascular necrosis	19(16.5)	13(17.3)	1	29(16)	3(33.3)	0.18	18(19.1)	14(14.6)	0.44	23(15.8)	9(20.5)	0.49
TIA	4(3.5)	4(5.3)	0.72	7(3.9)	1(11.1)	0.33	3(3.2)	5(5.2)	0.72	5(3.4)	3(6.8)	0.39
DVT	19(16.5)	10(13.3)	0.68	29(16)	0(0)	0.36	13(13.8)	16(16.7)	0.69	24(16.4)	5(11.4)	0.48
CVA	12(10.4)	13(17.3)	0.19	22(12.2)	3(33.3)	0.1	13(13.8)	12(12.5)	0.83	17(11.6)	8(18.2)	0.31
MI	4(3.5)	1(1.3)	0.65	5(2.8)	0(0)	1	4(4.3)	1(1)	0.21	4(2.7)	1(2.3)	1
**Digital gangrene**	3(2.6)	3(4)	0.68	6(3.3)	0(0)	1	**0(0)**	**6(6.2)**	**0.03**	5(3.4)	1(2.3)	1
Venous Thrombosis	24(20.9)	13(17.3)	0.58	36(19.9)	1(11.1)	1	16(17)	21(21.9)	0.47	30(20.5)	7(15.9)	0.66
Arterial Thrombosis	19(16.7)	19(25.3)	0.19	34(18.9)	4(44.4)	0.08	18(19.4)	20(20.8)	0.86	26(17.9)	12(27.3)	0.2
Pneumonia	45(39.1)	28(37.3)	0.88	72(39.8)	1(11.1)	0.16	30(31.9)	43(44.8)	0.07	55(37.7)	18(40.9)	0.73
Pyelonephritis	13(11.3)	5(6.7)	0.32	17(9.4)	1(11.1)	0.6	9(9.6)	9(9.4)	1	14(9.6)	4(9.1)	1
**Sepsis**	**4(3.5)**	**10(13.3)**	**0.02**	14(7.7)	0(0)	1	**3(3.2)**	**11(11.5)**	**0.05**	12(8.2)	2(4.5)	0.53
Abscess	10(8.7)	11(14.7)	0.24	21(11.6)	0(0)	0.6	6(6.4)	15(15.6)	0.06	15(10.3)	6(13.6)	0.58
Cellulitis	12(10.4)	10(13.3)	0.64	21(11.6)	1(11.1)	1	10(10.6)	12(12.5)	0.82	13(8.9)	9(20.5)	0.06
Opportunistic infections	32(27.8)	21(28)	1	51(28.2)	2(22.2)	1	22(23.4)	31(32.3)	0.2	40(27.4)	13(29.5)	0.85
**Prednisone**	38(33)	30(40)	0.36	160(88.4)	8(88.9)	1	**25(26.6)**	**43(44.8)**	**0.01**	53(36.3)	15(34.1)	0.86
Plaquenil	113(98.3)	74(98.7)	1	178(98.3)	9(100)	1	92(97.9)	95(99)	0.62	145(99.3)	42(95.5)	0.14
Cytotoxic tx	74(64.3)	43(57.3)	0.36	112(61.9)	5(55.6)	0.74	59(62.8)	58(60.4)	0.77	85(58.2)	32(72.7)	0.11
Azathioprine	35(30.4)	26(34.7)	0.63	58(32)	3(33.3)	1	28(29.8)	33(34.4)	0.54	47(32.2)	14(31.8)	1
Cyclophosphamide	25(21.7)	16(21.3)	1	40(22.1)	1(11.1)	0.69	17(18.1)	24(25)	0.29	30(20.5)	11(25)	0.54
**Methotrexate**	**25(21.7)**	**7(9.3)**	**0.03**	31(17.1)	1(11.1)	1	18(19.1)	14(14.6)	0.44	25(17.1)	7(15.9)	1
**Mycophenolate**	36(31.3)	27(36)	0.53	61(33.7)	2(22.2)	0.72	27(28.7)	36(37.5)	0.22	**42(28.8)**	**21(47.7)**	**0.03**
Rituximab	6(5.2)	6(8)	0.54	12(6.6)	0(0)	1	5(5.3)	7(7.3)	0.77	12(8.2)	0(0)	0.07
Belimumab	1(1.1)	0(0)	1	1(0.7)	0(0)	1	0(0)	1(1.3)	0.5	1(0.9)	0(0)	1

SCLE: subacute cutaneous lupus erythematosus, OBS: Organic brain syndrome, MM: mononeuritis multiplex, ACL, abnormal anti-cardiolipin antibody test, SS: Sjögren’s Syndrome, PHT: Pulmonary hypertension, LFT: Liver function tests, GI: gastrointestinal, TIA: transitory ischemic attack, DVT: deep vein thrombosis, MI: myocardial infarction. Associations were estimated with the chi-square test or Fisher’s exact test as appropriate.

Supplemental Table 3. Clinical, laboratory and disease activity associations at time of visit according to anti-Ro52 antibody type.

	Anti-Ro52											
	'Classic'			Nt			Ex4			γCT		
Clinical	Negative n = 113	Positive n = 74	p.value	Negative n = 178	Positive n = 9	p.value	Negative n = 93	Positive n = 94	p.value	Negative n = 147	Positive n = 40	p.value
Weight, kg	75(22)	78(20)	0.68	77(22)	78(15)	0.90	78(17)	76(22)	0.66	78(20.0)	74.6(18.6)	0.37
SBP, mmHg	124.9(17.06)	124.5(20.95)	0.89	124.9(18.8)	120.4(15.39)	0.42	124.8(17.78)	124.6(19.55)	0.94	125(18.99)	123.8(17.48)	0.72
**DBP, mmHg**	**70.9(11.15)**	**75.3(10.92)**	**0.01**	72.7(11.36)	71.6(8.95)	0.73	**70.6(11.28)**	**74.6(10.9)**	**0.02**	72.4(11.62)	73.2(9.8)	0.66
HGB, mg/dl	12.5(1.42)	12.3(1.82)	0.40	12.4(1.61)	12.5(1.35)	0.81	12.6(1.34)	12.3(1.8)	0.16	12.5(1.54)	12.1(1.74)	0.14
WBC, k/mm^3	6(2.21)	5.9(2.61)	0.86	6(2.4)	5.9(1.84)	0.96	5.8(2.05)	6.1(2.65)	0.31	6(2.44)	5.6(2.12)	0.27
Neutrophils, k/mm^3	3.9(1.92)	3.9(2.03)	0.88	3.9(1.97)	4(1.59)	0.94	3.6(1.63)	4.1(2.18)	0.10	4(1.98)	3.7(1.89)	0.44
**Neutrophils, %**	62.7(12.93)	63.7(13.89)	0.65	63.2(13.42)	61.9(9.58)	0.78	**60.8(11.98)**	**65.2(14.07)**	**0.04**	63.2(13.25)	62.9(13.62)	0.91
**Lymphocytes,** k/mm^3	1.6(0.75)	1.4(0.72)	0.17	1.5(0.73)	1.8(0.89)	0.51	**1.6(0.77)**	**1.4(0.69)**	**0.04**	1.5(0.77)	1.4(0.6)	0.33
**Lymphocytes, %**	27.2(11.54)	25(11.53)	0.27	26.2(11.66)	27.6(8.16)	0.73	**28.9(10.77)**	**24.1(11.8)**	**0.01**	26.3(11.66)	26.4(11.33)	0.93
Platelets, k/mm^3	249.6(75.92)	266.7(92.98)	0.19	255.6(83.95)	272(72.56)	0.53	254.1(73.39)	258.7(92.46)	0.71	256.2(88.78)	257.4(60.87)	0.92
Creatinine, mg/dl	0.9(0.28)	1(0.73)	0.47	0.9(0.51)	0.9(0.27)	0.80	0.9(0.27)	1(0.66)	0.41	0.9(0.54)	1(0.36)	0.12
UrRBC, per/field	1(2.26)	1(2.28)	0.87	0.9(2.04)	2.4(4.83)	0.40	0.9(1.98)	1.1(2.51)	0.68	0.9(1.8)	1.3(3.43)	0.51
**UrPr/Cr**	0.2(0.41)	0.4(1.28)	0.11	**0.3(0.9)**	**0.1(0.05)**	**0.01**	0.1(0.43)	0.4(1.14)	0.10	0.3(0.98)	0.1(0.26)	0.14
**hsCRP, mg/L**	4.5(6.83)	4.3(7.05)	0.91	4.5(7.04)	3.3(4.81)	0.64	4.2(6.4)	4.6(7.48)	0.81	**4.9(7.29)**	**1.3(1.32)**	**<0.001**
**ESR, mm/hr**	28.9(27.37)	35.7(28.25)	0.11	31.7(28.2)	29.6(19.88)	0.78	28.3(26.93)	34.9(28.51)	0.11	**28.9(25.07)**	**41.2(34.67)**	**0.04**
**C3, mg/dl**	125.4(39.91)	121.5(39.08)	0.51	122.9(39.18)	142.1(44.27)	0.24	**130.2(41.81)**	**117.7(36.33)**	**0.03**	**127.5(40.2)**	**110.9(34.46)**	**0.01**
C4 mg/dl	23.6(10.08)	23.7(11.02)	0.95	23.3(10.36)	30.1(10.23)	0.08	25(9.97)	22.3(10.76)	0.08	24.2(10.72)	21.8(9.21)	0.16
Anti-DNA, AU	32.5(111.73)	52.2(141.51)	0.32	40.2(125.54)	41.4(105.3)	0.97	28.1(103.34)	52.3(141.51)	0.19	31(99.51)	73.3(185.86)	0.17
IgG ACL, GPL	9.2(12.54)	7.2(4.02)	0.12	8.5(10.31)	6.4(3.47)	0.16	9.5(13.58)	7.3(4.08)	0.14	7.6(7.53)	11.4(15.93)	0.14
**IgM ACL, MPL**	8.1(4.08)	9(9.11)	0.45	**8.6(6.69)**	**6.7(2.12)**	**0.04**	7.9(4.15)	9(8.3)	0.27	8.5(7.12)	8.4(4.03)	0.94
IgA ACL, APL	4.5(3.91)	4.7(3.44)	0.72	4.6(3.76)	5(3.12)	0.73	4.9(4.16)	4.3(3.24)	0.32	4.5(3.48)	4.8(4.52)	0.69
**RVVT, seconds**	40.6(15.76)	37(9.4)	0.06	**39.4(13.88)**	**33.1(3.57)**	**<0.001**	39.7(16.1)	38.6(10.79)	0.62	**39.9(14.98)**	**36.2(5.57)**	**0.02**
**SLEDAI**	2.1(2.69)	2.8(3.05)	0.09	**2.4(2.91)**	**1.3(1)**	**0.02**	**1.8(2.24)**	**2.9(3.28)**	**0.01**	2.2(2.8)	3.1(2.96)	0.08
**LAI score (0-3)**	0.6(0.6)	0.7(0.7)	0.09	0.6(0.65)	0.4(0.33)	0.06	**0.5(0.58)**	**0.8(0.68)**	**0.01**	0.6(0.67)	0.6(0.53)	0.55
A: fatigue	0(0)	0(0)	NaN	0(0)	0(0)	NaN	0(0)	0(0)	NaN	0(0)	0(0)	NaN
**B: rash**	0.2(0.34)	0.3(0.53)	0.08	0.2(0.43)	0.2(0.36)	0.88	**0.2(0.27)**	**0.3(0.53)**	**0.03**	0.2(0.45)	0.2(0.31)	0.92
C: joints	0.2(0.32)	0.2(0.32)	0.45	0.2(0.33)	0.2(0.25)	0.84	0.2(0.33)	0.2(0.31)	0.30	0.2(0.32)	0.2(0.33)	0.64
D: serositis	0(0)	0(0.14)	0.32	0(0.09)	0(0)	0.32	0(0)	0(0.12)	0.32	0(0)	0(0.19)	0.32
A: neurological	0(0.27)	0(0.12)	0.36	0(0.23)	0(0)	0.07	0(0.28)	0(0.15)	0.62	0(0.25)	0(0)	0.07
**B: renal**	0.2(0.51)	0.3(0.65)	0.23	**0.2(0.58)**	**0(0)**	**<0.001**	**0.1(0.46)**	**0.3(0.65)**	**0.04**	0.2(0.57)	0.2(0.56)	0.77
C: pulmonary	0	0	NaN	0	0	NaN	0	0	NaN	0	0	NaN
**D: heme**	0.1(0.3)	0.1(0.28)	0.85	**0.1(0.3)**	**0(0)**	**<0.001**	0.1(0.32)	0.1(0.26)	0.92	**0.1(0.32)**	**0(0.11)**	**0.02**
**Prednisone (mgs)**	2.6(4.34)	3.7(6.93)	0.23	**3.2(5.63)**	**1.1(2.2)**	**0.03**	**1.6(2.56)**	**4.5(7.08)**	**<0.001**	3.1(5.91)	2.7(3.84)	0.60

SBP: Systolic blood pressure, DBP: Diastolic blood pressure, UrRBC: urinary red blood cells, UrPr/Cr: Urinary protein/creatinine ratio, hsCRP: high-sensitivity c-reactive protein, ESR: erythrocyte sedimentation rate, ACL: anti-cardiolipin antibodies, RVVT: Dilute Russell Viper Venom Time, SLEDAI: Systemic Lupus Erythematosus Disease Activity Index, LAI: Lupus Activity Index. Variables are summarized as mean (SD). Comparisons between groups were done using Student's T test.
